# The landscape of kinase fusions in cancer

**DOI:** 10.1038/ncomms5846

**Published:** 2014-09-10

**Authors:** Nicolas Stransky, Ethan Cerami, Stefanie Schalm, Joseph L. Kim, Christoph Lengauer

**Affiliations:** 1 Blueprint Medicines, Cambridge, Massachusetts 02142, USA

## Abstract

Human cancer genomes harbour a variety of alterations leading to the deregulation of key pathways in tumour cells. The genomic characterization of tumours has uncovered numerous genes recurrently mutated, deleted or amplified, but gene fusions have not been characterized as extensively. Here we develop heuristics for reliably detecting gene fusion events in RNA-seq data and apply them to nearly 7,000 samples from The Cancer Genome Atlas. We thereby are able to discover several novel and recurrent fusions involving kinases. These findings have immediate clinical implications and expand the therapeutic options for cancer patients, as approved or exploratory drugs exist for many of these kinases.

## Abstract (web summary)

Kinases activated by gene fusions represent potentially important targets for the development of cancer drugs. Here, the authors develop a method for detecting gene fusion events in RNA sequencing data from The Cancer Genome Atlas and identify several novel recurrent fusions involving kinases.

## Graphical abstract

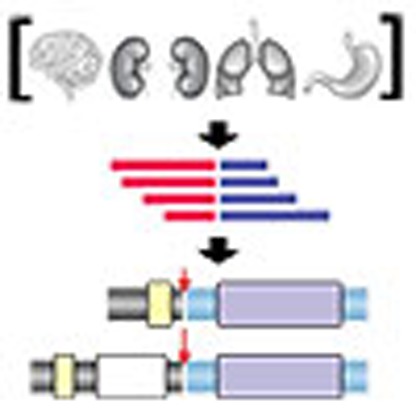

Kinases activated by gene fusions represent an important class of oncogenes associated with both hematopoietic malignancies and solid tumours. They are produced by translocations or other chromosomal rearrangements, and their protein products often represent ideal targets for the development of cancer drugs. For example, imatinib induces remission in leukaemia patients who are positive for *BCR*–*ABL1* fusions. More recently, crizotinib and ceritinib have produced significant clinical benefit results in patients with lung carcinomas and mesenchymal tumours harbouring anaplastic lymphoma kinase (*ALK*) fusions^1^,^2^.

Advances in massively parallel sequencing technologies have enabled the genomic characterization of large panels of tumours through the study of their DNA. While such studies have helped to identify numerous point mutations and small insertion/deletions in genes driving tumorigenesis, our understanding of the landscape of gene fusions in solid tumours is incomplete. There are now several thousand cancer transcriptomes, assessed through RNA-seq, publicly available.

Here we describe a computational pipeline for the identification of gene fusions that we applied to the entire RNA-seq data set from The Cancer Genome Atlas (TCGA). We identify several novel and recurrent fusions involving kinases that very likely play a role in cancer. These discoveries not only go beyond augmenting our understanding of the genomic landscape of cancers, but they also have immediate implications for cancer diagnosis and therapy.

## Results

### Hallmarks of kinase fusions in solid tumours

We first assembled a computational pipeline to collect evidence for all possible gene fusions and then focused our analyses on the fusions involving a kinase. Given the very large number of samples, we prioritized the sensitivity of the fusion detection pipeline (Methods) while retaining the ability to exclude false-positive calls by filtering out fusions present in normal samples as well as highly recurrent fusions that were detected by the algorithm at improbable frequencies. We then focused our detailed analysis exclusively on recurrent (*n*≥2 across all cancer types), putatively functional kinase fusions (Supplementary Fig. 1).

**Figure 1:**
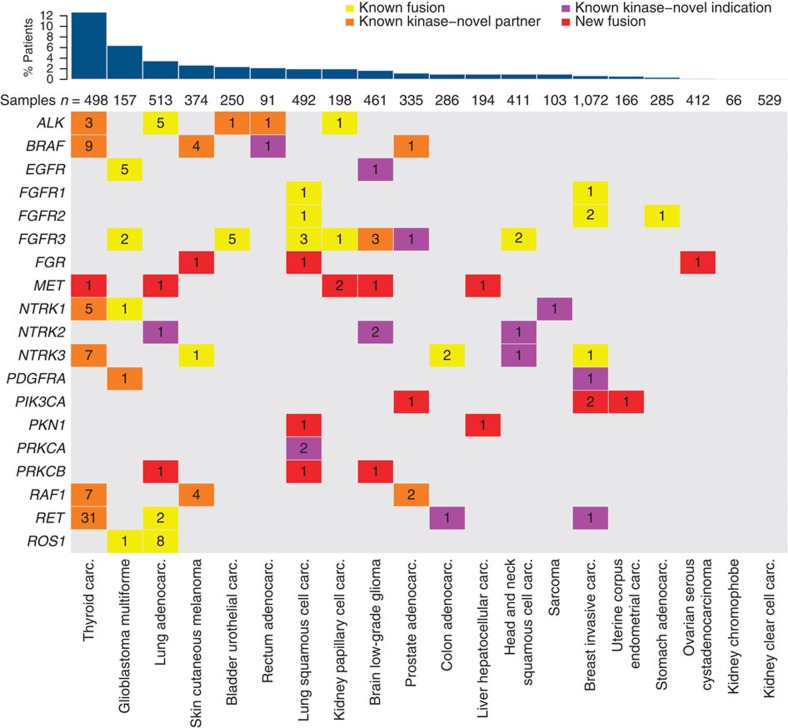
Landscape of recurrent kinase fusions in solid tumours. Tumour types are indicated at the bottom and ordered by frequency of samples harbouring recurrent fusions (%; bar chart at the top). For each gene, the number of fusions found in TCGA samples is displayed in the matrix and coloured by the type of novelty. Yellow denotes kinase fusions that have been described previously in this particular indication; orange denotes kinase fusions for which one or more partner genes are novel but the indication is not; purple denotes a novel indication for a particular kinase fusion regardless of the identity of the partner gene; red denotes novel, recurrent kinase fusions.

We comprehensively surveyed gene fusions across 20 solid tumour types (Supplementary Table 1, Supplementary Data 1) and identified several broad contours within the landscape of kinase fusions (Fig. 1). First, as has been observed for point mutations, the proportion of samples harbouring kinase fusions was markedly different between cancer types, reflecting differences in the aetiology of these tumours. For instance, sarcoma samples showed the highest frequency of kinase fusions (0.57 fusions per sample), consistent with the current understanding that a large fraction of sarcomas harbour specific translocations, but only 12% of those were recurrent kinase fusions (Supplementary Fig. 1). Other tumour types, including thyroid cancer and glioblastoma, showed a lower frequency of kinase fusions on average (0.19 and 0.23 fusions per sample, respectively) but a relatively high proportion of these were recurrent fusions (67% and 36%, respectively), suggesting a more prominent role of kinase fusions in these cancers. Conversely, some cancer types, for example, clear cell and chromophobe renal cell carcinoma, showed a very low frequency of kinase fusions with no instances of recurrence (Supplementary Fig. 1). Overall, we detected recurrent kinase fusions in 3.0% of the samples, and all cancers except clear cell and chromophobe renal cell carcinoma harboured recurrent kinase fusions (0–12.9% of samples per cancer type, median=2.1%).

Second, our pipeline was able to recapitulate most known translocation events in cancer (*ALK*, *BRAF*, *EGFR*, *FGFR1*, *2* and *3*, *NTRK1*, *2* and *3*, *PDGFRA*, *PRKCA*, *RAF1*, *RET*, *ROS1*). Interestingly, we identified new tumour types harbouring such fusions and discovered several novel fusion partners for these kinases. We also detected several low-frequency, pan-cancer kinase fusion events, for example in the neurotrophic tyrosine receptor kinases *NTRK1*, *NTRK2* and *NTRK3*, that drive tumorigenesis in a small fraction of multiple cancers, regardless of tissue type (Fig. 1).

Third, we identified several novel and recurrent kinase fusions that very likely play a role in cancer, such as those involving the *MET* proto-oncogene and *PIK3CA* (phosphatidylinositol-4,5-bisphosphate 3-kinase, catalytic subunit alpha). These *bona fide* oncogenes have not been shown previously to be activated by fusion events. Our analysis also uncovered novel, recurrent fusions in kinases with no known tumorigenic genomic alterations (that is, feline Gardner–Rasheed sarcoma viral oncogene homologue, *FGR* and protein kinase *N1, PKN1*), potentially resulting in active and oncogenic fusion proteins. Finally, we discovered a recurrent fusion in sarcoma encoding the non-catalytic portion of *TRIO* kinase, which resulted in the upregulation of the transcription of telomerase reverse transcriptase (*TERT*) in those tumours.

### Kinases known to be involved in gene fusions

*ALK* fusions, including *EML4*–*ALK*, *TFG*–*ALK* and *STRN*–*ALK*, have been identified in multiple cancer types, including lung adenocarcinoma^3^, colorectal^4^, breast^5^, renal cell^6^, renal medullary^7^ and thyroid cancers^8^. Consistent with previous studies, we detected *EML4*–*ALK* fusions in ~1% (5/513) of lung adenocarcinoma samples, multiple *ALK* fusions, including a single *STRN*–*ALK* fusion, in thyroid cancer (3/498) and one in papillary renal carcinoma. We also found several novel *ALK* fusion events, including a *TPM1*–*ALK* fusion in bladder cancer, a *SMEK2*–*ALK* fusion in rectal adenocarcinoma and a *GTF2IRD1*–*ALK* fusion in thyroid cancer (Fig. 1, Supplementary Fig. 2), adding weight to the emerging notion that known driver events in certain tumours can also play a role in other tumour types, regardless of histology.

We also identified multiple c-ros oncogene 1 (*ROS1*) fusions, including *ROS1* fusions in 8/513 lung adenocarcinomas, all of which have been previously described^9^,^10^,^11^,^12^. In addition, we detected a *CEP85L*–*ROS1* fusion in a glioblastoma tumour sample (Fig. 1, Supplementary Fig. 3). A similar fusion was recently reported in a single angiosarcoma sample^13^.

*RET* proto-oncogene fusions have been identified previously in both lung adenocarcinoma^4^,^14^ and thyroid cancer^15^. Consistent with these studies, we observed recurrent *CCDC6*–*RET* fusions in thyroid cancer^15^ but also identified several *RET* fusions with novel partners, including *AKAP13*, *FKBP15*, *SPECC1L* and *TBL1XR1* (Fig. 1, Supplementary Fig. 4). Three of these 5′ fusion partners contain dimerization-competent coiled-coil motifs within the coding region, while the fourth, *TBL1XR1,* contains a LisH (Lis-homology) motif, which is similarly capable of dimerization^16^ and therefore likely leading to RET activation. In addition, we detected previously identified *RET* fusions in new tumour indications, including a single *CCDC6*–*RET* fusion in colon adenocarcinoma and a single *ERC1*–*RET* fusion in breast cancer. In total, we observed *RET* fusions in four of the 20 cancer types analysed, providing a therapeutic rationale for the use of *RET* inhibitors in multiple patient subpopulations.

*BRAF* (v-raf murine sarcoma viral oncogene homologue B) fusions have also been described previously in multiple cancer types, including prostate cancer^17^, melanoma^18^, radiation-induced thyroid cancer^19^ and pediatric low-grade gliomas^20^. Consistent with these studies, we identified a broad range of cancer types harbouring *BRAF* fusions, including prostate, melanoma and thyroid. We also detected a single *TRIM24*–*BRAF* fusion in rectal adenocarcinoma. Interestingly, the *BRAF* fusions in melanoma are exclusive of other known oncogenic events such as *BRAF* and *NRAS* mutations. Many of the specific fusions we report here, including *AGK*–*BRAF*^19^, *SND1*–*BRAF*^21^, *MACF1*–*BRAF*^20^, *TAX1BP1*–*BRAF* and *CDC27*–*BRAF*^18^, have been previously identified. A number of *BRAF* fusions are, however, novel (Fig. 1, Supplementary Fig. 5), including *ATG7*–*BRAF* in melanoma, as well as *ZC3HAV1*–*BRAF* and *FAM114A2*–*BRAF* in thyroid cancer. These three fusions encode 5′ protein partners that contribute coiled-coil (CC) or zinc-finger dimerization motifs, which likely produce constitutively activated BRAF dimers capable of driving tumorigenesis and poorly sensitive to RAF inhibitors, but sensitive to inhibition downstream, through MEK (mitogen-activated protein kinase kinase 1 and 2) inhibition for instance^18^. Other novel *BRAF* fusions identified do not encode protein partners with obvious dimerization motifs. However, these fusions all remove at least the first eight exons of BRAF, which has previously been shown to promote BRAF dimerization, independent of activated RAS (rat sarcoma viral oncogene homologues) or other mechanisms of BRAF dimerization^22^. These fusions similarly seem capable of promoting tumorigenesis.

Consistent with previous studies^17^,^23^, we also found recurrent *RAF1* (also known as *CRAF*) fusions in various tumour types (Fig. 1, Supplementary Fig. 6). In addition to known tumour occurrences (four fusions in melanoma, two fusions in prostate adenocarcinoma), we identified *AGGF1*–*RAF1* fusions in seven papillary thyroid carcinoma samples (1.4%). This is remarkable not only because *RAF1* fusions have never been identified in this cancer type before, but also because all seven examples involved a novel partner gene, *AGGF1* (*angiogenic factor with G patch and FHA domains 1*). AGGF1 contains an N-terminal coiled–coil dimerization motif likely to activate RAF1 in a fashion similar to *BRAF* fusions, by forming constitutively activated, RAF inhibitor resistant, RAF1 dimers. *AGGF1*–*RAF1* fusions appear not to be limited to thyroid cancers, as we also found a single *AGGF1*–*RAF1* fusion in prostate cancer.

We observed a broad distribution of the fibroblast growth factor receptors *FGFR1*, *FGFR2* and *FGFR3* fusions—in particular *FGFR3*–*TACC3* fusions—across eight of the 20 tumour types analysed (Fig. 1). This is consistent with recent studies, which identified recurrent *FGFR* family fusions in multiple cancer types^24^,^25^. We also detected a single *FGFR3*–*TACC3* fusion in a novel indication, papillary renal carcinoma, and a novel *FGFR3*–*ELAVL3* fusion in low-grade glioma (Supplementary Fig. 7). Similar to *RET* and *NTRK1*–*3* (see below), fusions involving *FGFR1*–*3* provide a therapeutic opportunity for current and future FGFR inhibitors in multiple patient subpopulations.

Recurrent fusions involving members of the *NTRK* family have been identified previously in congenital fibrosarcoma^26^, human secretory breast carcinoma^27^ and papillary thyroid cancer^28^, which represent clinical indications for which currently available non-kinase-targeted treatment options are usually adequate. However, recurrent *NTRK1* and *NTRK2* fusions have also been recently identified in diseases which represent significant unmet medical needs, including glioblastoma^29^, cholangiocarcinoma^30^ and pediatric high-grade glioma^24^. Consistent with previous studies, we observed recurrent *NTRK1* and *NTRK3* fusions in papillary thyroid cancer and glioblastoma, but also identified a number of novel *NTRK2* fusions in head and neck squamous cell carcinoma (*PAN3*–*NTRK2*), low-grade glioma (*AFAP1*–*NTRK2*) and lung adenocarcinoma (*TRIM24*–*NTRK2*) (Fig. 1, Supplementary Fig. 8). In addition, we observed a known *NTRK1* fusion (*TPM3*–*NTRK1*) in sarcoma, previously described only in thyroid cancer^31^. Across all tumour types analysed, *NTRK1–3* fusions were observed at low frequency in 9 of the 20 cancer types analysed, providing a therapeutic opportunity for the use of pan-NTRK inhibitors in multiple patient populations.

Protein kinase C fusions have recently been described in papillary glioneuronal tumours^32^ and benign fibrous histiocytoma^33^. We found two new occurrences of *PRKCA* (protein kinase C, alpha) fusions in lung squamous cell carcinoma and three *PRKCB* (protein kinase C, beta) fusions in lung squamous cell carcinoma, lung adenocarcinoma and low-grade glioma (Fig. 1, Supplementary Fig. 9a,c). In one instance, *PRKCA* was fused with *IGF2BP3* (insulin-like growth factor 2 messenger RNA (*mRNA*) binding protein 3), an mRNA binding protein present in the nucleus and the cytoplasm. The functional domains of IGF2BP3, such as a nucleotide binding/RNA recognition domain, are intact in the fusion; however their contribution to PRKCA activation is unclear. The second fusion, *TANC2*–*PRKCA*, encodes only the first two exons of *TANC2* (tetratricopeptide repeat, ankyrin repeat and CC containing 2), which contain no annotated structural domain or motif. In both cases, however, N-terminal truncation of PRKCA removes the autoinhibitory pseudosubstrate segment, possibly leading to a constitutively activated kinase in the absence of a functional fusion partner. In addition, we noticed a tendency towards overexpression of *PRKCA* in these two fusion-harbouring samples (*PRKCA* mRNA expression *z*-scores: 6.8 and 2.6 in the samples harbouring the *TANC2*–*PRKCA* and *IGF2BP3*–*PRKCA* fusions, respectively; Supplementary Fig. 9b). These data suggest that *PRKCA* fusions are potential oncogenic events in lung squamous cell carcinoma, leading to overexpression as well as constitutive activation of PRKCA. In the same fashion, *PRKCB* fusions truncate the N-terminal part of the protein containing the autoinhibitory domain and are predicted to activate this kinase (Supplementary Fig. 9c). Taken together, these data suggest an emerging critical role for protein kinase C alpha and beta in the tumorigenesis of non-small cell lung cancer.

### Novel fusions involving known oncogenes

**Figure 2:**
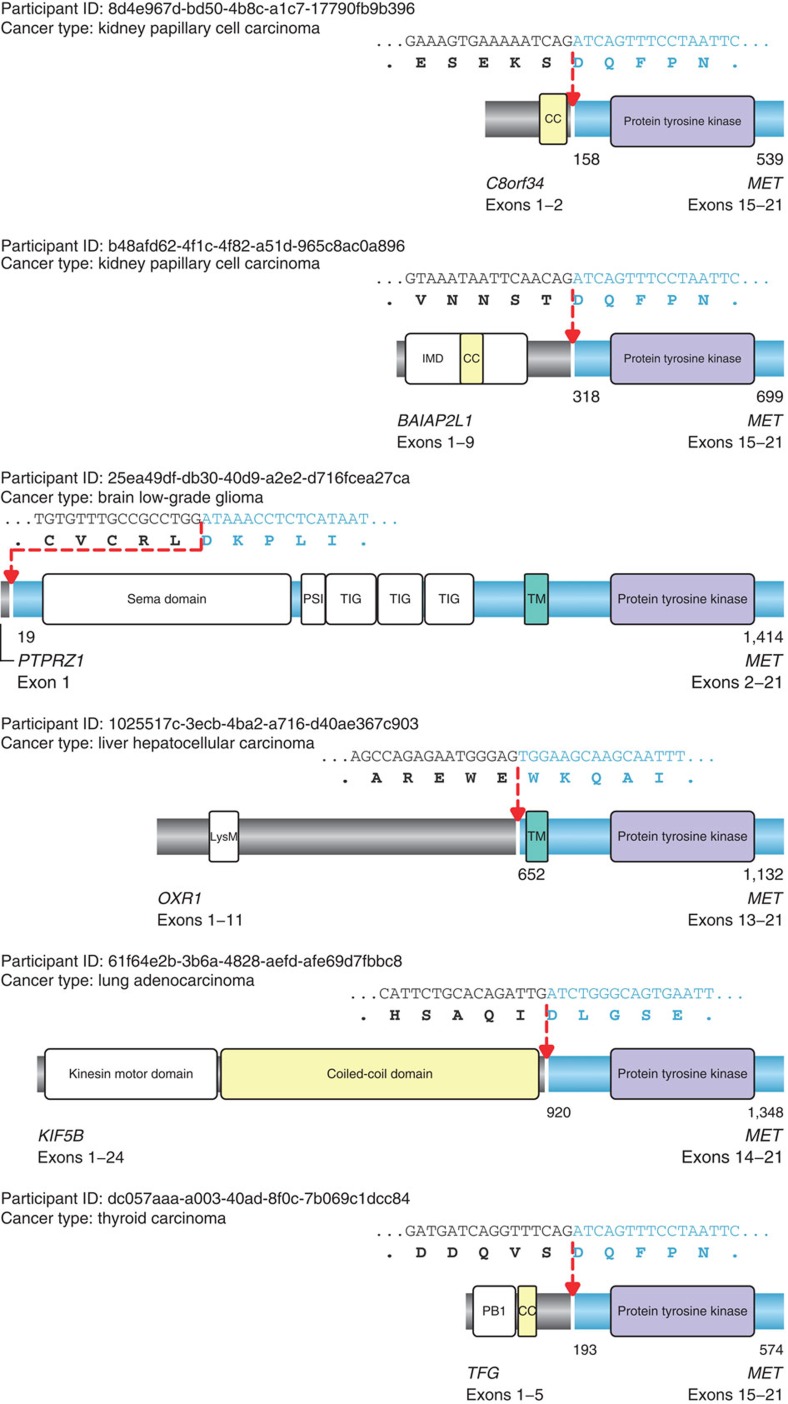
Structural details of *MET* fusions. Novel fusions of *MET* proto-oncogene in five cancer types. Protein domains, sample details, cancer type, gene partners, as well as genomic and amino-acid sequence are indicated for each of the six predicted *MET* kinase fusion proteins. In addition, the protein coordinates of the fusion breakpoints and the total amino-acid length of the fusion protein are noted under each protein structure. The protein tyrosine kinase domains are coloured in purple, the transmembrane domains (TM) are indicated in teal, CC dimerization domains are indicated in yellow, whereas other domains are left in white. Fusion breakpoints are delineated by red arrows. IMD, IRSp53/MIM homology domain; PSI, Plexin repeat domain; TIG, IPT/TIG immunoglobulin-like domain; LysM, lysin motif domain; PB1, Phox and Bem1p domain.

The *MET* proto-oncogene is implicated in a variety of cancers, particularly in papillary renal cell carcinoma where a number of somatic mutations have been described^34^. Anecdotally, a transforming *TPR*–*MET* fusion was previously generated *in vitro* via carcinogen-induced chromosomal rearrangement fusing the dimerization domain of TPR to the kinase domain of the MET receptor tyrosine kinase^35^. Here, we report for the first time in primary tumour samples recurrent translocation events involving *MET*. Two in-frame *MET* fusions in papillary renal carcinoma, *BAIAP2L1*–*MET* and *C8orf34*–*MET,* were detected with predicted protein products containing amino-terminal dimerization domains fused to the intracellular domain of MET. BAIAP2L1 contains a CC region while C8orf34 contains a regulatory subunit of cAMP-dependent protein kinase, both of which act as dimerization motifs (Fig. 2). Notably, *BAIAP2L1* was recently described as a 3′ fusion partner for *FGFR3* in bladder cancer^36^, incorporating the same CC region as the 5′ fusion described here. We also identified single *MET* fusions in four other cancers: low-grade glioma, hepatocellular carcinoma, lung adenocarcinoma and thyroid carcinoma. In at least two out of these four cases (*KIF5B*–*MET* in lung adenocarcinoma and *TFG*–*MET* in thyroid papillary carcinoma), the predicted chimeric protein follows the classic activation paradigm, fusing dimerization motifs to an intact kinase domain. These results are remarkable because *MET* is a known oncogene that has not previously been implicated in translocation events. This mechanism could account for a significant fraction of total *MET* oncogenic activation events and therefore represents druggable intervention opportunities for patients with these tumours.

**Figure 3:**
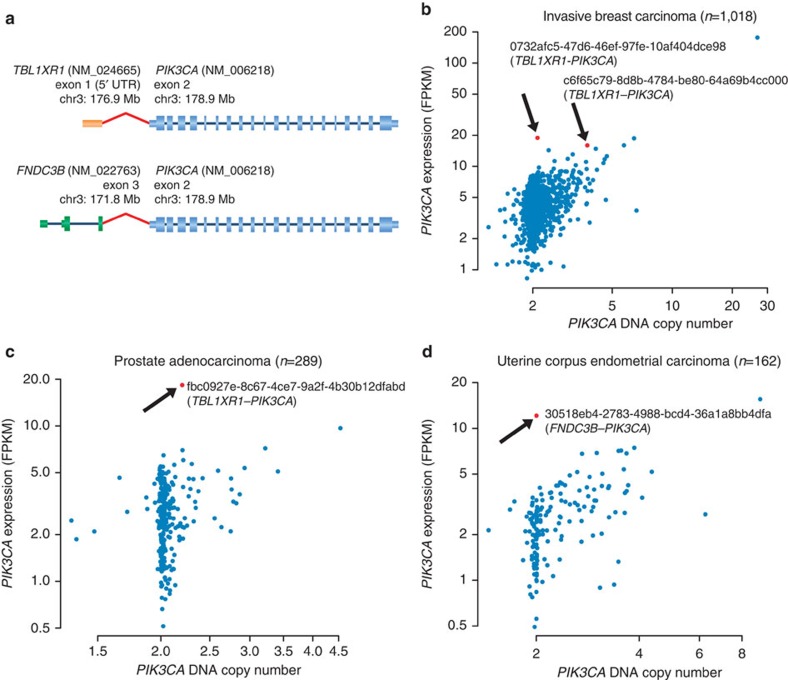
*PIK3CA* promoter fusions. Novel fusions of *PIK3CA* in three cancer types. (**a**) Genomic structure of *TBL1XR1*–*PIK3CA* and *FNDC3B*–*PIK3CA* fusions. In both cases, the entire coding sequence of *PIK3CA* is present in the predicted fusion sequence. RefSeq IDs, exon numbers and genomic coordinates are indicated. Exon 1 of *TBL1XR1* (5′UTR) is fused to exon 2 of *PIK3CA* (5′UTR). In the case of *FNDC3B*–*PIK3CA*, exon 3 of *FNDC3B* is fused to the 5′UTR of *PIK3CA*. (**b**–**d**) Scatter plots of *PIK3CA* DNA copy number versus mRNA expression across all TCGA samples for which both data types are available. Samples harbouring a *PIK3CA* fusion are depicted in red (along with TCGA sample ID) and show a high expression of *PIK3CA* relative to the other samples. FPKM, fragments per kilobase of transcript per million mapped reads.

Mutations and, to a lesser extent, increased copy numbers in another prevalent oncogene, *PIK3CA*, have been characterized in diverse cancers. While activating missense mutations in *PIK3CA* have been described as frequently as 50% in endometrial cancers, 30% in breast invasive carcinomas and 20% in colorectal as well as head and neck cancers^37^, this gene has not been implicated in activating fusion events. We found two *TBL1XR1*–*PIK3CA* fusions in 1,072 breast cancer samples, and a single occurrence of the same gene fusion in prostate adenocarcinoma (1/335). In addition, one *FNDC3B*–*PIK3CA* fusion was found in uterine corpus endometrial carcinoma (1/166) (Fig. 1, Supplementary Figs 10 and 11). The nucleotide sequence of the fusion transcripts suggested that the complete wild-type sequence of *PIK3CA* was expressed in all four cases, with the partner gene contributing only the 5′UTR (untranslated region), and thereby driving overexpression of *PIK3CA* (Fig. 3a). Indeed, in all samples where we detected *PIK3CA* translocations, and where *PIK3CA* was not amplified, *PIK3CA* mRNA expression levels were the highest within the respective tumour types (Fig. 3b–d). Interestingly, *TBL1XR1* is thought to regulate the expression of nuclear hormone receptor co-repressors^38^, and both tissue types in which *TBL1XR1*–*PIK3CA* fusions were found (invasive breast carcinoma and prostate cancer), are hormone driven and ranked among the highest for *TBL1XR1* mRNA expression across all normal tissues (Supplementary Fig. 12). These results strongly suggest that *PIK3CA* overexpression is driven by its fusion partner, and that *PIK3CA* promoter fusions are an additional oncogenic mechanism to be considered for expanding the use of targeted therapies such as PI3K, AKT or mTOR inhibitors.

### Novel recurrent fusions

In addition to fusions involving known oncogenes, we found several novel and recurrent fusions involving kinases that have not been previously directly linked to cancer (Fig. 1, Supplementary Data 2). One of these kinases was *FGR*, a member of the Src family of protein tyrosine kinases. Here, we show for the first time that genetic events can lead to *FGR* overexpression in primary tumour samples. We found three *WASF2*–*FGR* fusions (in lung squamous carcinoma, ovarian serous cystadenocarcinoma and skin cutaneous melanoma), harbouring the exact same breakpoints in all cases (Supplementary Figs 13 and 14). The *WASF2* and *FGR* genes are located very proximally on the short arm of chromosome 1, and the fusion presumably results from a tandem repeat that puts their coding regions in close proximity (Supplementary Fig. 15a,b). Somewhat similarly to *TBL1XR1*–*PIK3CA*, the promoter and 5′UTR of *WASF2* are fused with the 5′UTR of *FGR*, leading to mis- or overexpression of the entire wild-type sequence of the protein. In all three cases, *FGR* mRNA expression in the samples harbouring a fusion was among the highest compared with all other tumours of that tissue type (Supplementary Fig. 15c–e). Even though *FGR* has not been genetically linked to cancer to date, it has been hypothesized that its expression could compensate for SRC inhibition^39^. Collectively, these data highlight a previously undocumented mechanism of genetic deregulation of a Src family member.

*PKN1* has been implicated in androgen-associated prostate carcinomas^40^ and in Wnt/β-catenin signalling in melanomas^41^. We detected fusions of *PKN1* in samples of squamous cell carcinoma of the lung and hepatocellular carcinoma (Fig. 1, Supplementary Fig. 16a). mRNA expression levels of both fusions are high within the respective tumour types (Supplementary Fig. 16b,c). Interestingly, the protein sequences contributed by the non-kinase fusion partners were very limited in both cases (three and five amino acids in *ANXA4*–*PKN1* and *TECR*–*PKN1*, respectively) and resulted in a truncated PKN1 protein product missing the PKN1 N terminus. This is notable because this protein region contains regulatory domains that suppress kinase activity in the absence of binding to Rho-GTP^42^. PKN1 also has a Caspase-3 cleavage site near the breakpoint of both fusions that normally results in activation of the kinase on cleavage^43^ (Supplementary Fig. 16a). Therefore, both of these fusions are potentially activating in the absence of any functional or structural contribution from the non-kinase fusion partners. It is attractive to speculate that both fusion events cause increased PKN1 expression and constitutive activation of the kinase, leading to enhanced cell proliferation.

## Discussion

**Figure 4:**
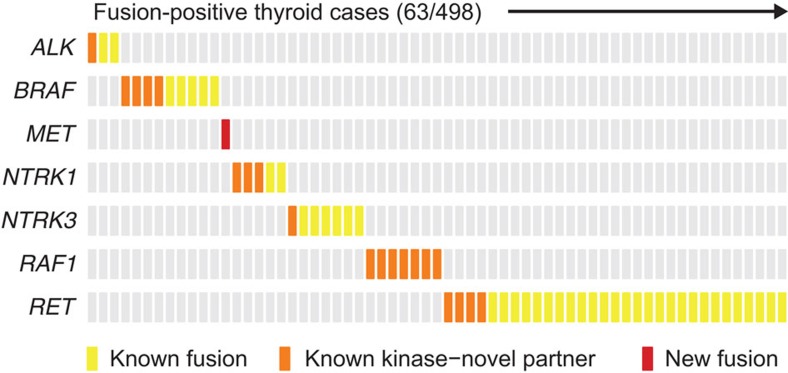
Mutual exclusivity of fusions in thyroid cancer. All samples harbouring a recurrent kinase fusion in any of the genes indicated on the left are displayed on the *x* axis. The type of fusion is depicted as a coloured box with the same color-coding scheme as in Fig. 1. Yellow denotes kinase fusions that have been described previously in thyroid cancer; orange denotes kinase fusions in which the partner gene is novel; red denotes novel recurrent kinase fusions. In all cases, the presence of a kinase fusion is exclusive of any other fusion involving kinases recurrently fused.

We describe here a pan-cancer analysis of the transcriptomes of nearly 7,000 tumours from TCGA that is specifically focused on kinase gene fusion events. Overall, 3.0% of tumour samples contained a likely oncogenic, recurrent kinase fusion (2.1% excluding thyroid cancer). The observed striking differences in the frequencies of kinase fusions across solid tumours are consistent with previous data on the relative contributions of diverse types of genetic aberrations to tumourigenesis. Certain tumour types, such as ovarian serous carcinoma, harbour a large number of somatic copy number alterations, but exhibit a relatively simple mutational profile^44^. Other tumour types such as melanoma carry predominantly somatic point mutations^45^. Consistent with these observations, our data suggest that certain cancers are heavily driven by kinase rearrangements. Notably, thyroid cancers have the highest frequency of recurrent kinase fusions (63/498, 13%), and all fusion events including *ALK*, *BRAF*, *MET*, *NTRK1*, *NTRK2*, *RAF1* and *RET* are mutually exclusive in this cancer type (Fig. 4). These data provide a strong genetic rationale that these alterations are driver events. In stark contrast, clear cell and chromophobe renal cell carcinoma have the lowest frequencies of kinase fusions, none of which were recurrent in our analysis of this data set (Supplementary Fig. 1).

Our study primarily aimed to identify recurrent, potentially oncogenic fusions involving kinases. In addition to rediscovering previously known recurrent kinase fusions, we identified new fusion partners for many genes (for example, *TPM1*–*ALK* in bladder cancer, *TBL1XR1*–*RET* in thyroid cancer, and so on). Our study also revealed new cancer types harbouring known fusions (for example, *BRAF* fusion in rectal adenocarcinoma, *FGFR3* fusion in prostate adenocarcinoma, *RET* fusions in colon adenocarcinoma and invasive breast carcinoma, *EGFR*–*SEPT14* in low-grade glioma, and so on). These discoveries not only go beyond simply augmenting our understanding of the genomic landscape of cancers, but they also have immediate implications for cancer diagnosis and therapy. First, our new findings justify a rapid reassessment of current protocols for targeted genomic profiling of patients, which are insufficient to detect these aberrancies, to cover therapeutically actionable fusion events across cancers. In addition, our findings will hopefully motivate both industrial and academic investigators interested in drug discovery to engage in the development of cancer drugs against targets that have not been considered previously because they might have represented an insufficient fraction of targetable events. Along these lines, our pan-cancer analysis revealed that although certain kinase fusions only occurred once within a tumour type, they are clearly recurrent when multiple tissue types are considered, supporting the emergence of innovative clinical trial designs such as the ‘basket’ trials. For example, we found six cancer types with a single fusion in a gene of the *NTRK* family, but altogether, we detected a total of 23 *NTRK1*, *NTRK2* and *NTRK3* fusions across nine tumour types. These data strongly suggest that gene fusions are one of the most prevalent mechanisms of oncogenic activation of this receptor tyrosine kinase family. *NTRK* fusions therefore represent a low frequency, pan-cancer event that nevertheless may account for a significant fraction of patients who could benefit from a pan-NTRK inhibitor.

Notably, this analysis uncovered several new aberrations that may drive tumourigenesis through constitutive activation of a kinase due to a fusion event. In particular, we found recurrent fusions of *MET* and *PIK3CA* that are both *bona fide* oncogenes commonly activated by gene amplification or point mutations. The activation of MET by fusion with a partner gene is most likely due to constitutive dimerization of the receptor. In contrast, *TBL1XR1*–*PIK3CA* fusions likely drive increased *PIK3CA* mRNA expression by juxtaposing the promoter region of the partner gene to the 5′ end of the intact *PIK3CA* coding sequence. Another example of such ‘promoter fusions’ is the recurrent *WASF2*–*FGR* fusion, which we found in three cancer types. We also observed fusions in some Ser/Thr kinases (for example, *PRKCA*, *PKN1*), where deletion of their regulatory N-terminal domain putatively leads to constitutive activation by de-repression of the kinase activity. Additional studies, expanding on our analyses, are necessary to uncover the mechanistic details of these newly described fusions and to further validate biologically the hypotheses we have put forward here.

Given the number of samples that we analysed (nearly 7,000 samples across 20 tumour types) and the observed frequencies of kinase fusion events across solid tumours, relatively few kinases appear to be recurrently fused in-frame with another gene while conserving an intact kinase domain (Supplementary Fig. 1). Therefore, a pure frequency threshold (that is, recurrence) allowed us to identify several new kinase fusion driver candidates. By excluding all kinases that were involved in only one fusion event across the entire data set, we disregarded several singletons that passed all other filtering criteria and showed the characteristics of functional fusions. With increasing numbers of tumours and additional cancer types being sequenced in the future, it is probable that some of these kinase fusions will appear more frequently and prove to be important drivers. One such example is *PRKACA* (protein kinase, *cAMP*-dependent, catalytic, alpha), which was recently shown to be fused in 100% (15/15) of fibrolamellar hepatocellular carcinoma^46^. Only one sample of fibrolamellar hepatocellular carcinoma was included in the TCGA data set that we analysed, and our study revealed that it indeed harboured the characteristic *DNAJB1*–*PRKACA* fusion. However, the majority of singleton gene fusions in this set are predicted to be passenger events, occurring as a consequence of chromothripsis or genomic instability. In addition, focal events such as gene amplification also contribute passenger events. This is likely the case for *ERBB2*, *PAK1*, *PDGFRA* and *RPS6KB1* (Supplementary Data 2 and 3)^47^.

**Figure 5:**
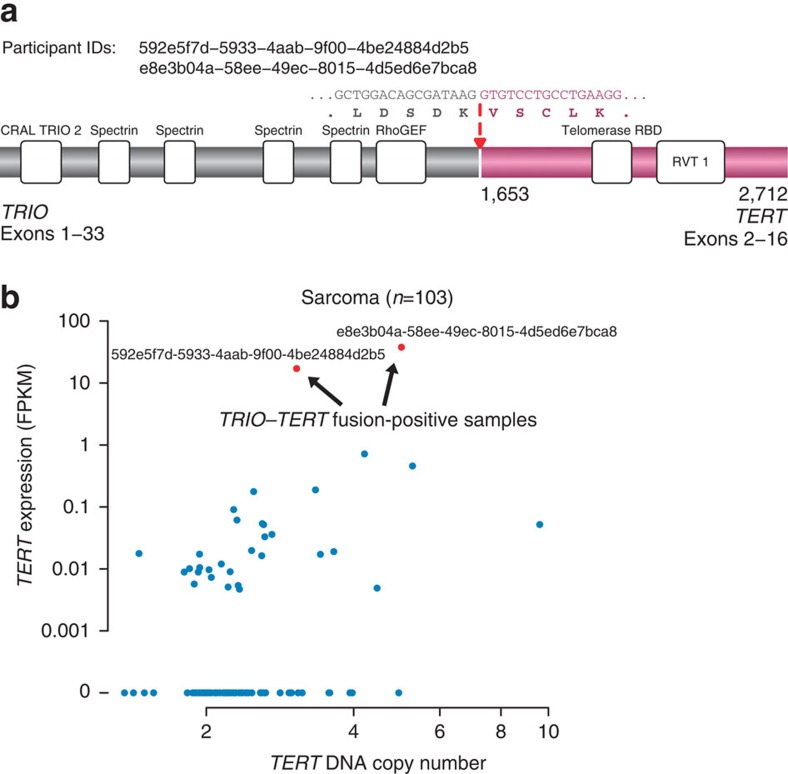
Novel *TRIO*–*TERT* fusions in liposarcoma samples. (**a**) Protein domains as well as genomic and amino-acid sequences are indicated for the *TRIO*–*TERT* fusion protein. Protein coordinates of the fusion breakpoints and the total amino-acid length of the fusion protein are noted under the protein structure; fusion breakpoint is delineated by a red arrow. CRAL TRIO 2, divergent CRAL/TRIO domain; Telomerase RBD, telomerase ribonucleoprotein complex–RNA binding domain; RVT 1: reverse transcriptase (RNA-dependent DNA polymerase). (**b**) Scatter plot of *TERT* DNA copy number versus mRNA expression across all TCGA samples for which both data types are available. Samples harbouring a *TERT* fusion are depicted in red (along with TCGA sample ID) and show a high expression of *TERT* relative to the other samples. FPKM: Fragments per kilobase of transcript per million mapped reads.

Finally, we predict that eventually some important, biologically meaningful fusions will be discovered that involve the non-enzymatic portion of kinases as partner genes. Although not the focus of this study, we describe here such an example: a recurrent fusion in two dedifferentiated liposarcoma samples (2/38; 5%) encoding the non-kinase portion of *TRIO*, which results in upregulation of its fusion partner *TERT* (Fig. 5a,b). Telomerase activity is a hallmark of many cancers, and two other genetic mechanisms of TERT reactivation have been described recently; both somatic mutations in the promoter of the *TERT* gene^48^ and DNA copy number gains of *TERT*^49^ were shown to activate its transcription. We observed that the two liposarcoma samples harbouring *TRIO*–*TERT* fusions display a *TERT* mRNA expression level ~100-fold higher than measured in samples without such a fusion. These findings raise the possibility that *TERT* fusions might represent an alternative mechanism for telomerase reactivation in cancers.

## Methods

### Overview

Briefly, fusions between any two genes were identified based on the number of chimeric reads (sequencing paired ends mapping to different genes) and split reads (spanning a fusion breakpoint), concordance between the strands of the reads and the genes involved in the putative fusion, and a number of filtering criteria to flag false positive and non-functional fusions. In addition, recurrent kinase fusions observed in a panel of 600 normal samples from TCGA and 1,800 normal samples from the Genotype–Tissue Expression (GTEx) project were also excluded from further analysis. Finally, all recurrent kinase fusions (*n*≥2) were manually reviewed to identify putative oncogenic drivers with distinctive characteristics of functional kinase fusions. In particular, the following features were required: presence of an intergenic junction between two exons, a predicted in-frame coding sequence and conservation of the complete kinase catalytic domain. Conversely, we excluded false positives from further analysis according to two main criteria: the presence of a homologous or repetitive sequence shared by the two fusion partners causing an alignment artifact, or the very high expression of one or both fusion partners.

### Origin of data

To comprehensively identify the landscape of kinase fusions in solid tumours, we analysed RNA-seq data from 20 solid tumour types in TCGA (6,893 samples, Supplementary Table 1 and Supplementary Data 1), including provisional TCGA data from sarcoma tumours (103 samples). All available unaligned RNA-seq data files (fastq files) were obtained from the Cancer Genomics Hub (CGHub) and loaded into our processing pipeline. In addition, the clinical data from all available tumour types were pulled from the TCGA FTP server ( https://tcga-data.nci.nih.gov/tcgafiles/ftp_auth/distro_ftpusers/anonymous/tumour).

### Fusion detection algorithm and filtering

Using the STAR v2.3.1q aligner^50^, RNA-seq data from each tumour sample was aligned to version hg19 of the human genome, while also providing transcriptome and splice junction annotations from the Gencode project v17 (ref. 51).

A different genome index was generated for each of the different read lengths encountered in the RNA-seq data. Runtime options passed to STAR to generate genome indexes included: STAR --runMode genomeGenerate, --genomeDir hg19_Gencode17.overhang<read-length>, --genomeFastaFiles <hg19 reference fasta files>, --sjdbGTFfile gencode.annotation.gtf and --sjdbOverhang <read-length–1>.

STAR was then used to produce alignments and was run with specific options including: STAR --readFilesIn <fastq_1.fq.gz> <fastq_2.fq.gz>, --readFilesCommand zcat, --genomeDir <STAR genome index>, --outSAMstrandField intronMotif, --outFilterIntronMotifs RemoveNoncanonicalUnannotated, --outReadsUnmapped None, --chimSegmentMin 15, --chimJunctionOverhangMin 15, --alignMatesGapMax 200000 and --alignIntronMax 200000.

Given the programme arguments described above, the output of the STAR aligner consisted of two separate files containing sequencing reads: aligned reads consistent with a normal reference (_Aligned.sam) and aligned reads indicative of a putative rearrangement (_Chimeric.sam). A fusion detection routine was then used to identify protein fusion candidates: using the python library HTSeq-0.5.4p1 (ref. 52) and transcriptome annotations from the Gencode project v17, the name-sorted ‘chimeric’ alignments in the output of the STAR aligner were examined to count the number of chimeric pairs (where each sequencing end aligns to a different gene) and reads split between two genes. Specific filters were then applied to improve specificity: the strands of the alignments were compared with the strands of the genes to keep only those consistent with a proper 5′→3′ fusion; putative fusions between homologous genes were discarded; putative fusions between genes and overlapping homologous genes were discarded as well. This procedure then returned the complete list of possible gene fusions in a given sample, modulo the alignment artifacts, along with the number of chimeric reads and split reads supporting each fusion.

Next, fusions were filtered based on the number of chimeric reads and paired reads supporting them: five chimeric reads or more were required when two or more split reads were present; 10 chimeric reads or more were required when only 1 split read was present; 20 chimeric reads or more were required when no split reads were detected.

Finally, the output of the fusion detection step above was filtered to further improve specificity. This step relied on the analysis of a large number of samples to filter out highly recurrent fusions that were detected at improbable frequencies within a cancer type: for instance, fusions detected in >95% of samples, or fusions where both gene partners are themselves involved in >1 fusions in >25% of samples, were flagged as putative false positives. In addition, recurrent kinase fusions observed using the same procedure in five samples or more in a panel of 647 normal samples from TCGA (downloaded from CGHub on 2014-03-10) and 1,750 normal samples from the GTEx project (downloaded from dbGaP project phs000424.v4.p1 on 2014-01-17, excluding all transformed and cancer cell lines) were also excluded. This allowed us to exclude a large number of library construction and alignment artifacts.

All recurrent kinase fusion candidates (*n*≥2 across cancer types) identified by this procedure were then manually reviewed in the Integrative Genomics Viewer^53^ to identify putative drivers with distinctive patterns of functional kinase fusions, and reject passenger and false-positive fusions. In particular, the following features were required for putative functional fusions: (1) presence of an intergenic junction (between two exons or between an exon and a cryptic exon); (2) a predicted in-frame coding sequence; (3) conservation of the full kinase catalytic domain.

Manual review was facilitated by the fact that passenger fusions could mainly be linked to the following: (1) the absence of a fusion protein coding sequence that was in-frame; (2) the kinase domain was absent or truncated from the predicted protein sequence; or (3) the kinase was found to be fused only once in all samples (non-recurrent fusion).

Conversely, we flagged false positives according to two main causes: (1) a homologous or repetitive sequence shared by the two fusion partners and causing an alignment artifact, or (2) very high expression of one or both fusion partners in a particular sample, causing the production of non-specific RNA chimera by *trans*-splicing. This process, occurring at the step of cDNA preparation through template switching by the reverse transcriptase^54^, produces multiple experimental artifacts that can appear like real fusions but lack a clear exon–exon breakpoint and generally are not supported by split reads.

### Functional annotation

Recurrent candidate fusion protein sequences were searched for structural domains against the Pfam database^55^. Particular attention was paid to breakpoints that occurred outside of structural domains of the kinase and the fusion protein partner. Fusion partner sequences were checked for presence of CC domains by the method of Lupas *et al.*^56^ Other dimerization or multimerization domains were checked within partner protein sequences using InterPro^57^.

### RNA-seq expression quantification

mRNA gene expression, measured in fragments per kilobase of mRNA per million mapped reads, were calculated for all CCDS transcripts^58^ in the Gencode v17 database^51^ using Cufflinks v2.1.1 (ref. 59) with the options that included the following: cufflinks --multi-read-correct, --GTF <gencode.annotation.CCDS.gtf>, --mask-file <gencode.annotation_chrM.gtf> <Aligned.bam>.

### Copy number data

Copy number data were downloaded from the 2014-04-16 release on the GDAC portal ( http://gdac.broadinstitute.org/).

## Author contributions

N.S., E.C. and C.L. designed the study. N.S. and E.C. developed the computational infrastructure, the methods and performed the analysis. N.S., E.C., S.S. and J.L.K. interpreted the results. N.S., E.C., S.S., J.L.K. and C.L. wrote the manuscript.

## Additional information

**How to cite this article**: Stransky, N. *et al.* The landscape of kinase fusions in cancer. *Nat. Commun.* 5:4846 doi: 10.1038/ncomms5846 (2014).

## Supplementary Material

Supplementary Figures and Supplementary Table Supplementary Figures 1-16 and Supplementary Table 1

Supplementary Data 1 List of all TCGA samples analyzed

Supplementary Data 2 List of all recurrent kinase fusions and sample ids

Supplementary Data 3 List of all non-recurrent and non-curated kinase fusions and sample ids
