# Supplementary material for: The landscape of kinase fusions in cancer
Source: Nat Commun. 2014 Sep 10;5:4846. doi: 10.1038/ncomms5846 (PMC4175590; doi:10.1038/ncomms5846)
Supplement: Supplementary Figures and Supplementary Table — Supplementary Figures 1-16 and Supplementary Table 1 [file ncomms5846-s1.pdf]

Supplementary Figures

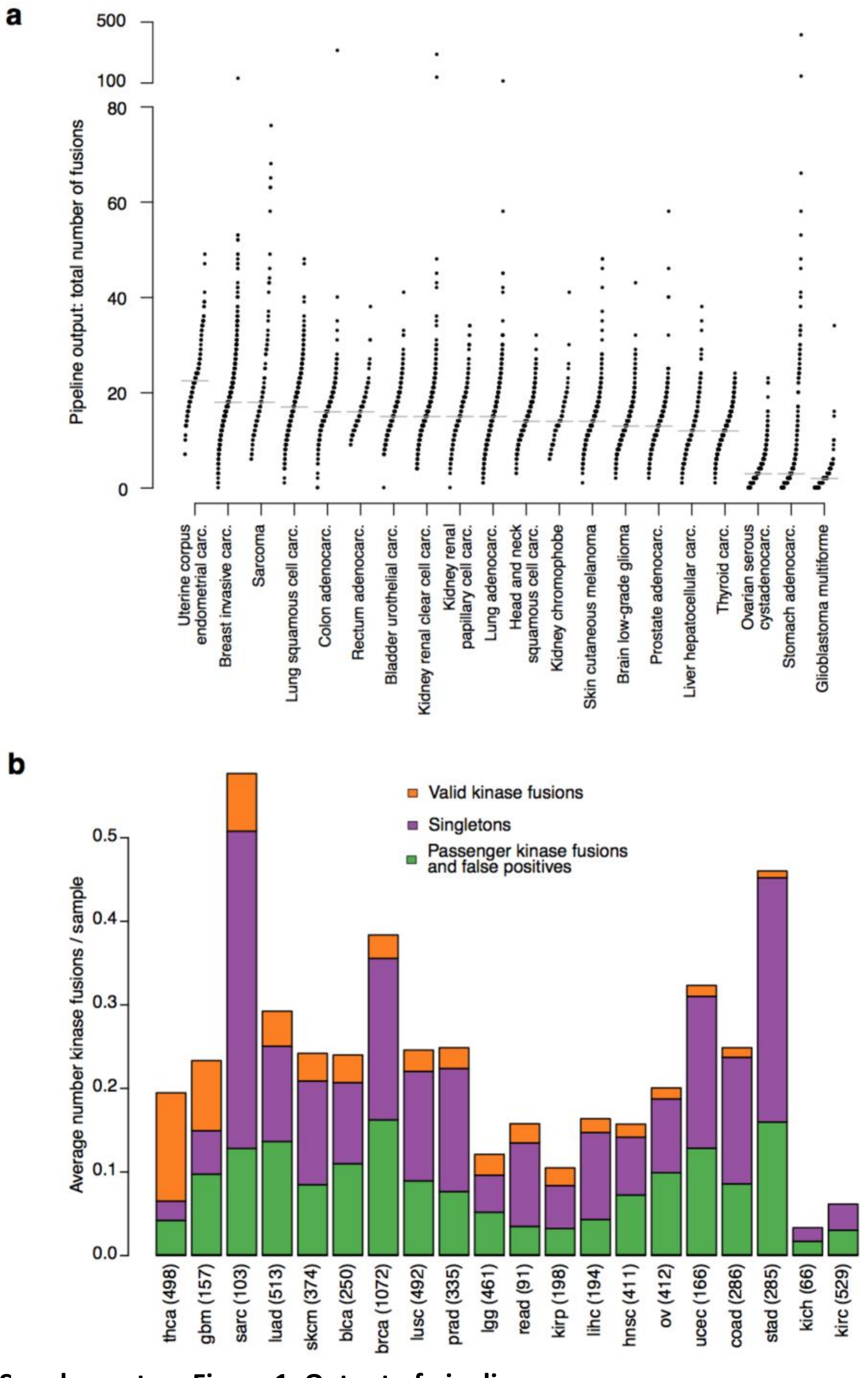

Supplementary Figure 1: Output of pipeline

a, Each dot corresponds to a tumor, with vertical position indicating the total number of fusions identified. Tumor types are ordered by their median number of fusions per sample, indicated by a grey horizontal line. **b**, For each tumor type, the average number of kinase fusions per sample is indicated and color-coded by category.

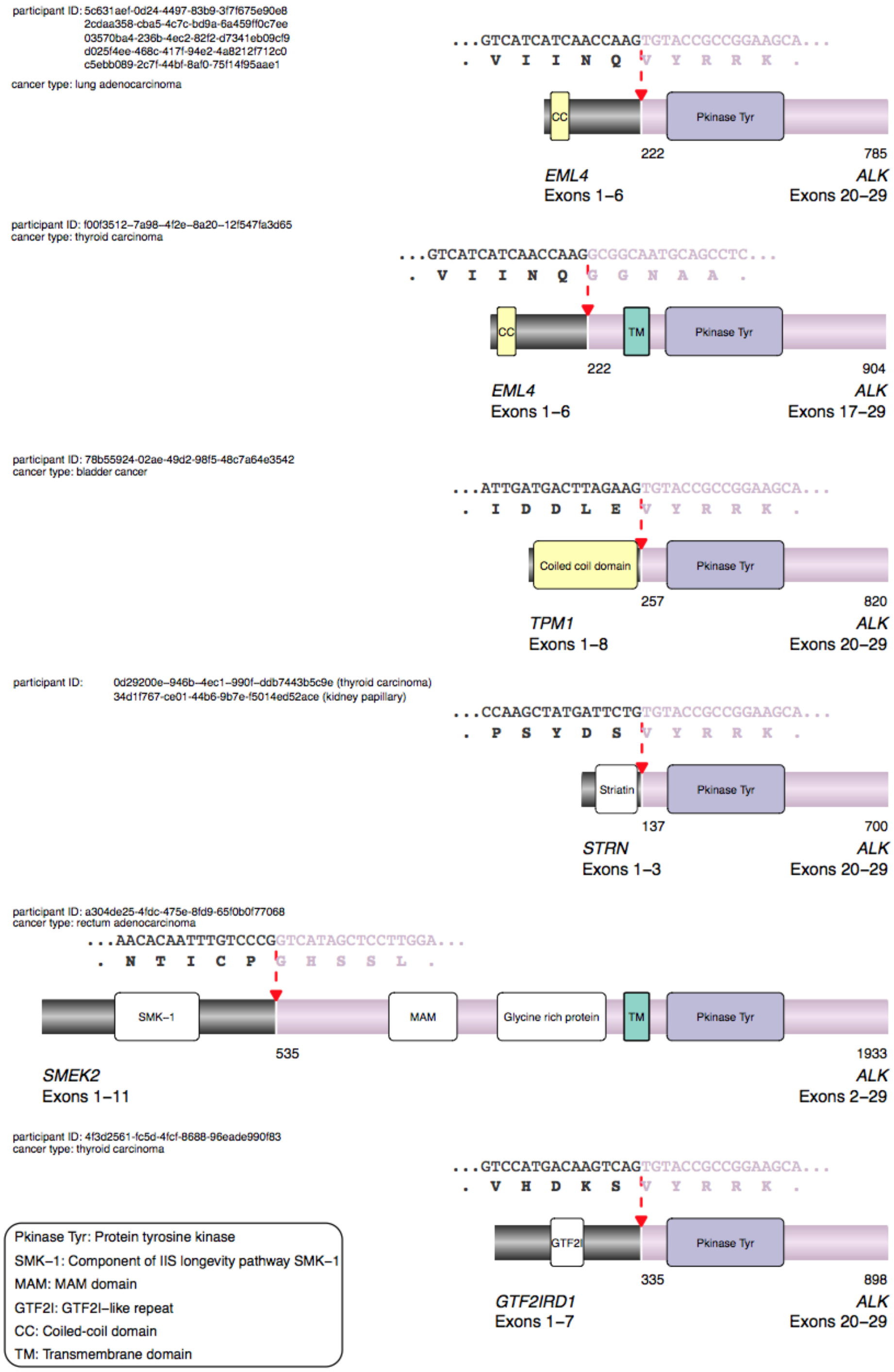

Supplementary Figure 2: *ALK* fusions

Protein domains, sample details, cancer type, gene partners, as well as genomic and amino acid sequence are indicated for each predicted kinase fusion proteins. In addition, the protein coordinates of the fusion breakpoints and the total amino acid length of the fusion protein are noted under each protein structure. The protein tyrosine kinase domains are colored in purple, the transmembrane domains (TM) are indicated in teal, coiled-coil (CC) dimerization domains are indicated in yellow, whereas other domains are left in white. Fusion breakpoints are delineated by red arrows.

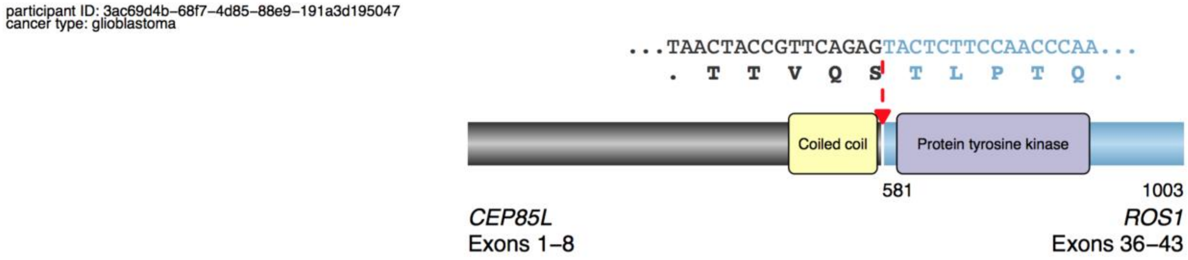

Supplementary Figure 3: ***ROS1*** **fusions**

Protein domains, sample details, cancer type, gene partners, as well as genomic and amino acid sequence are indicated for each predicted kinase fusion proteins. In addition, the protein coordinates of the fusion breakpoints and the total amino acid length of the fusion protein are noted under each protein structure. The protein tyrosine kinase domain is colored in purple, the coiled-coil (CC) dimerization domain is indicated in yellow. Fusion breakpoint is delineated by a red arrow.

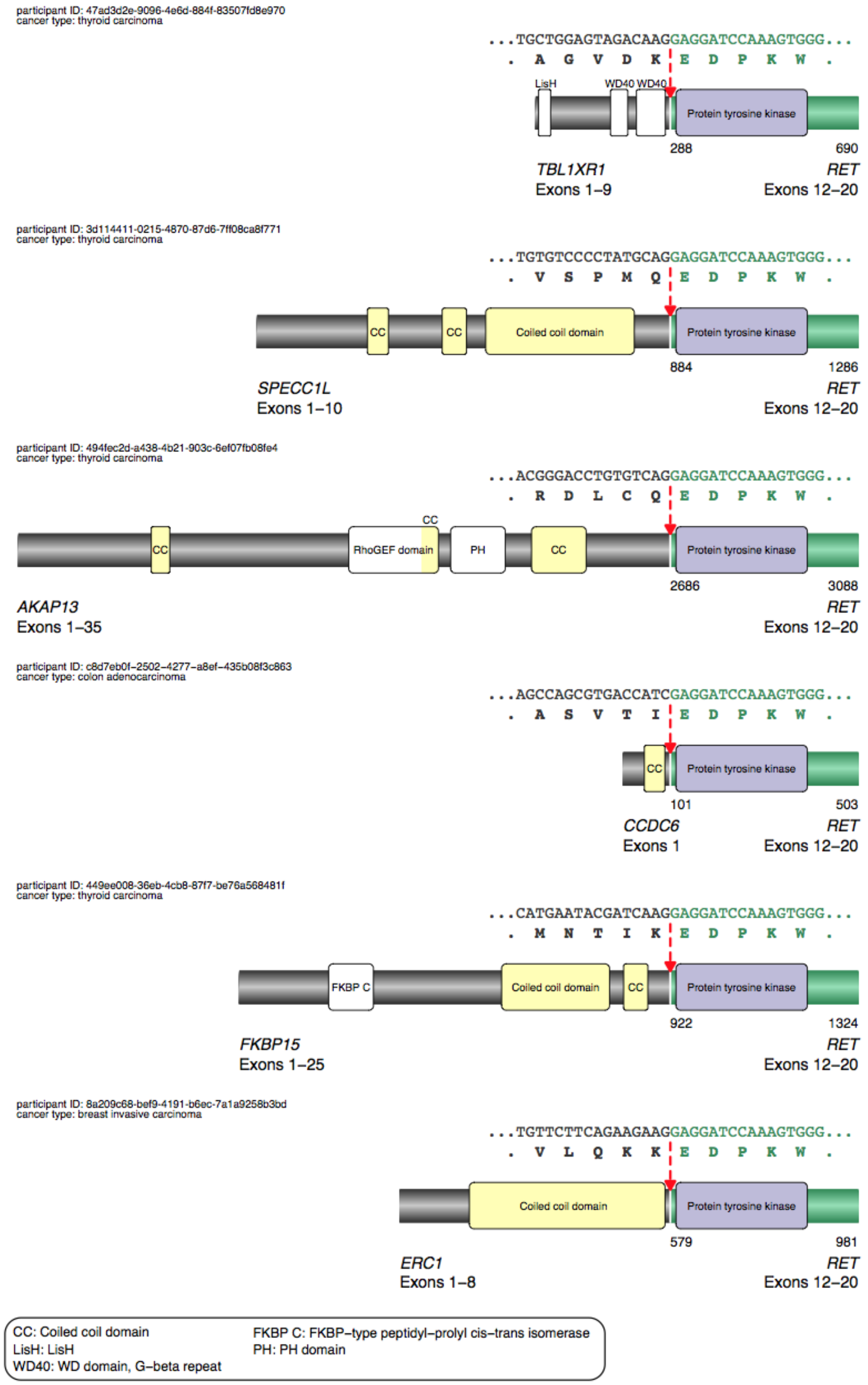

Supplementary Figure 4: *RET* fusions

Protein domains, sample details, cancer type, gene partners, as well as genomic and amino acid sequence are indicated for each predicted kinase fusion proteins. In addition, the protein coordinates of the fusion breakpoints and the total amino acid length of the fusion protein are noted under each protein structure. The protein tyrosine kinase domains are colored in purple, the transmembrane domains (TM) are indicated in teal, coiled-coil (CC) dimerization domains are indicated in yellow, whereas other domains are left in white. Fusion breakpoints are delineated by red arrows.

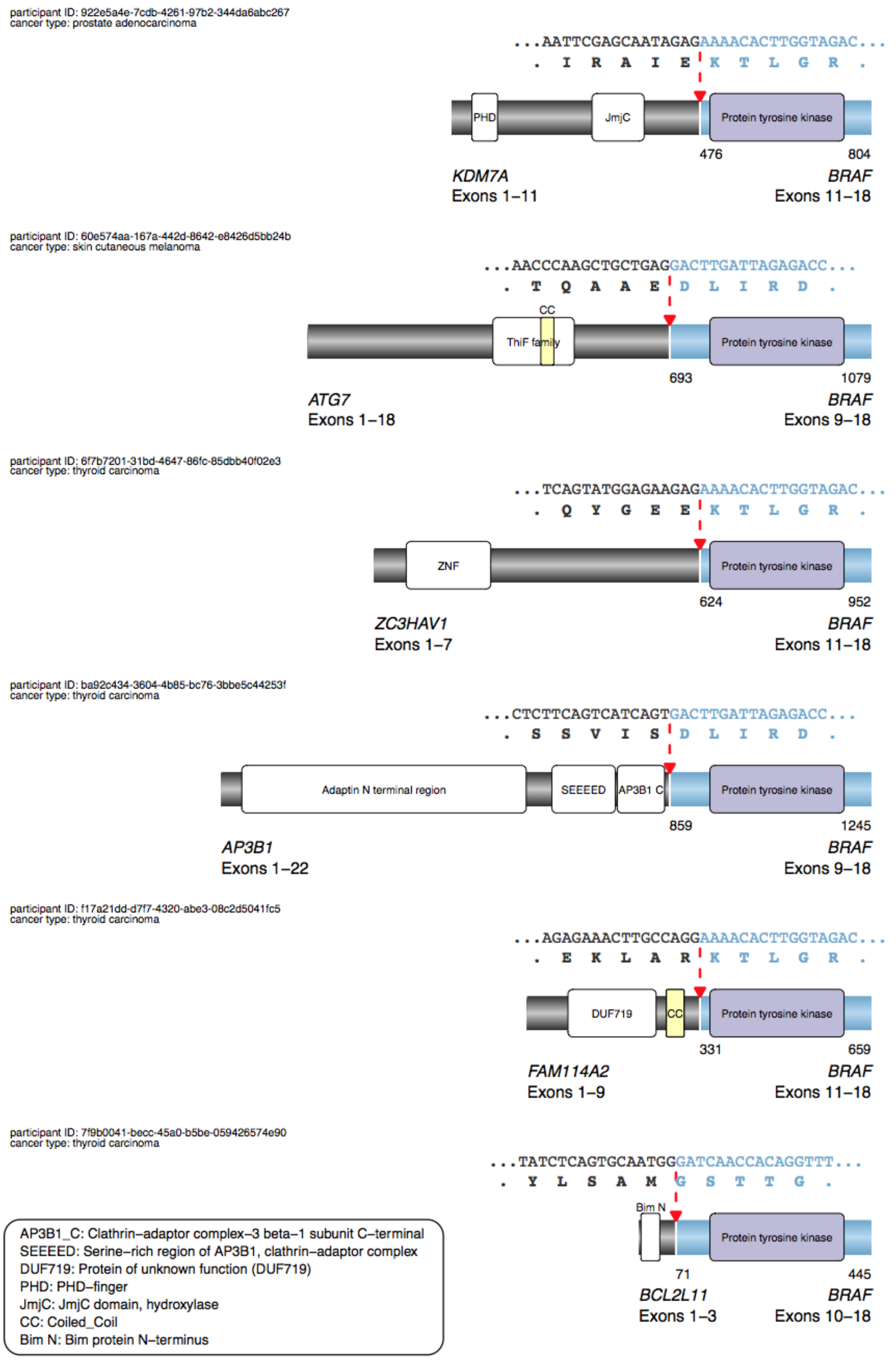

Supplementary Figure 5: *BRAF* fusions

Protein domains, sample details, cancer type, gene partners, as well as genomic and amino acid sequence are indicated for each predicted kinase fusion proteins. In addition, the protein coordinates of the fusion breakpoints and the total amino acid length of the fusion protein are noted under each protein structure. The protein tyrosine kinase domains are colored in purple, the transmembrane domains (TM) are indicated in teal, coiled-coil (CC) dimerization domains are indicated in yellow, whereas other domains are left in white. Fusion breakpoints are delineated by red arrows.

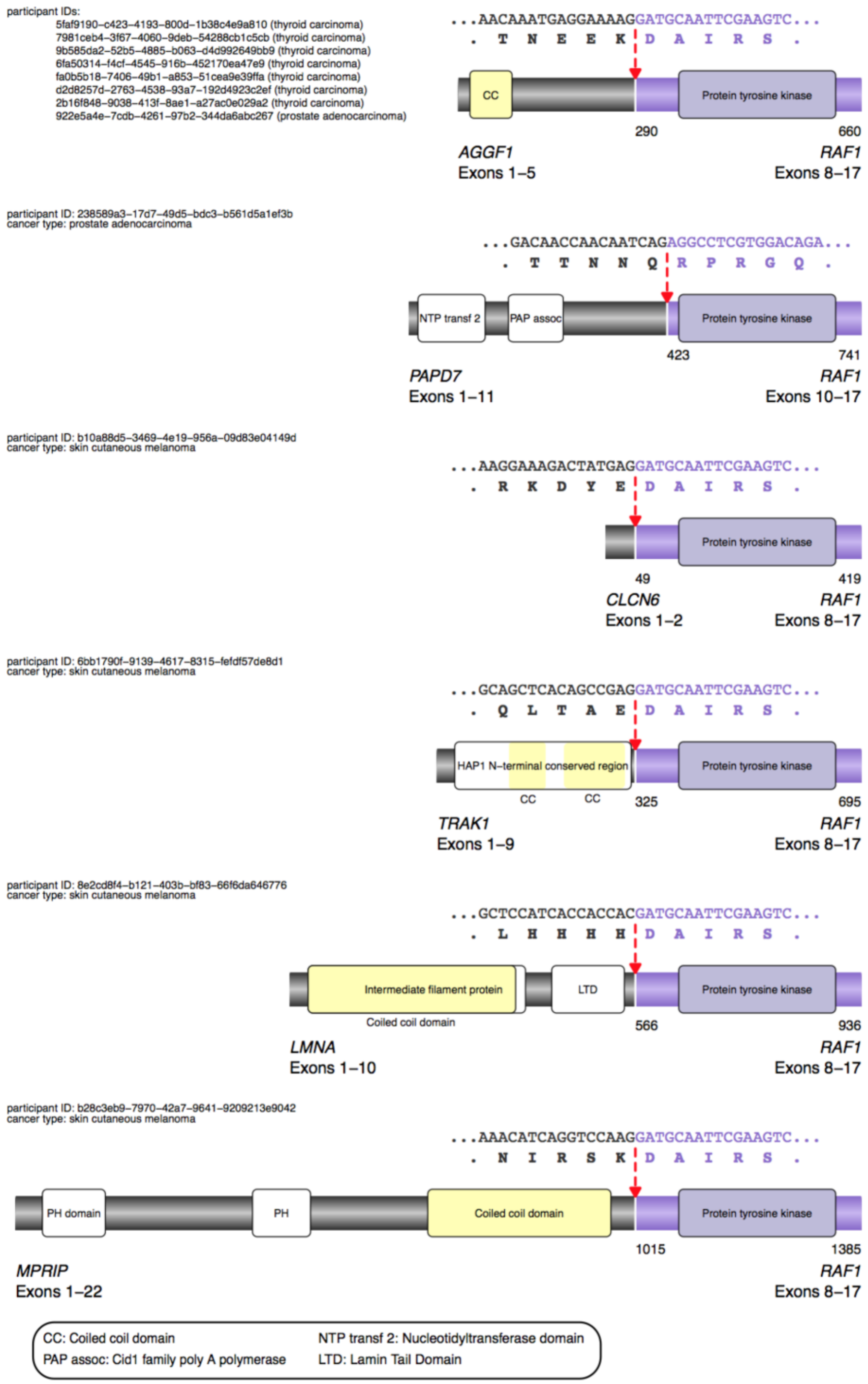

Supplementary Figure 6: *RAF1* fusions

Protein domains, sample details, cancer type, gene partners, as well as genomic and amino acid sequence are indicated for each predicted kinase fusion proteins. In addition, the protein coordinates of the fusion breakpoints and the total amino acid length of the fusion protein are noted under each protein structure. The protein tyrosine kinase domains are colored in purple, the transmembrane domains (TM) are indicated in teal, coiled-coil (CC) dimerization domains are indicated in yellow, whereas other domains are left in white. Fusion breakpoints are delineated by red arrows.

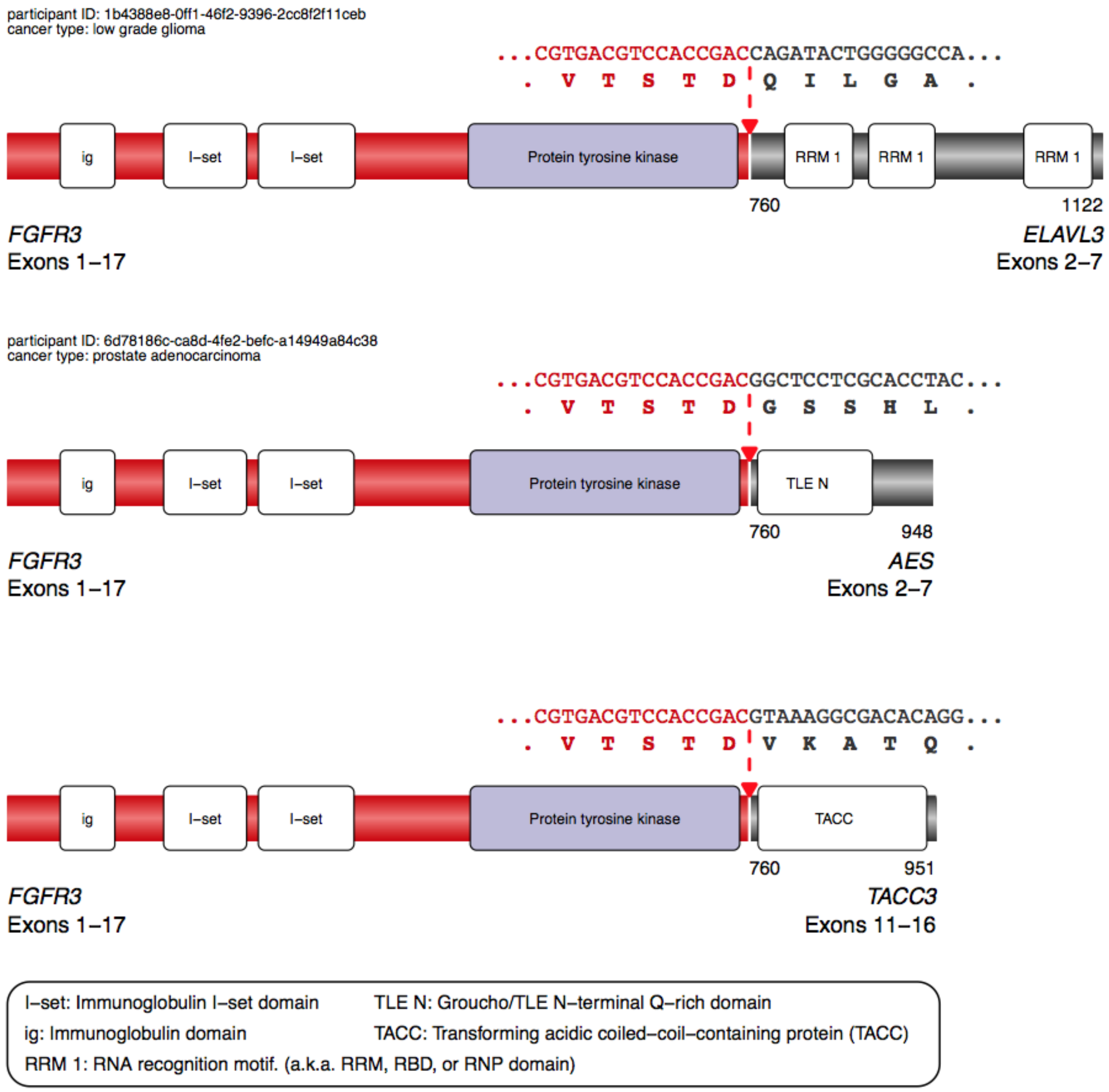

Supplementary Figure 7: *FGFR3* fusions

Protein domains, sample details, cancer type, gene partners, as well as genomic and amino acid sequence are indicated for each predicted kinase fusion proteins. In addition, the protein coordinates of the fusion breakpoints and the total amino acid length of the fusion protein are noted under each protein structure. The protein tyrosine kinase domains are colored in purple, the transmembrane domains (TM) are indicated in teal, coiled-coil (CC) dimerization domains are indicated in yellow, whereas other domains are left in white. Fusion breakpoints are delineated by red arrows.

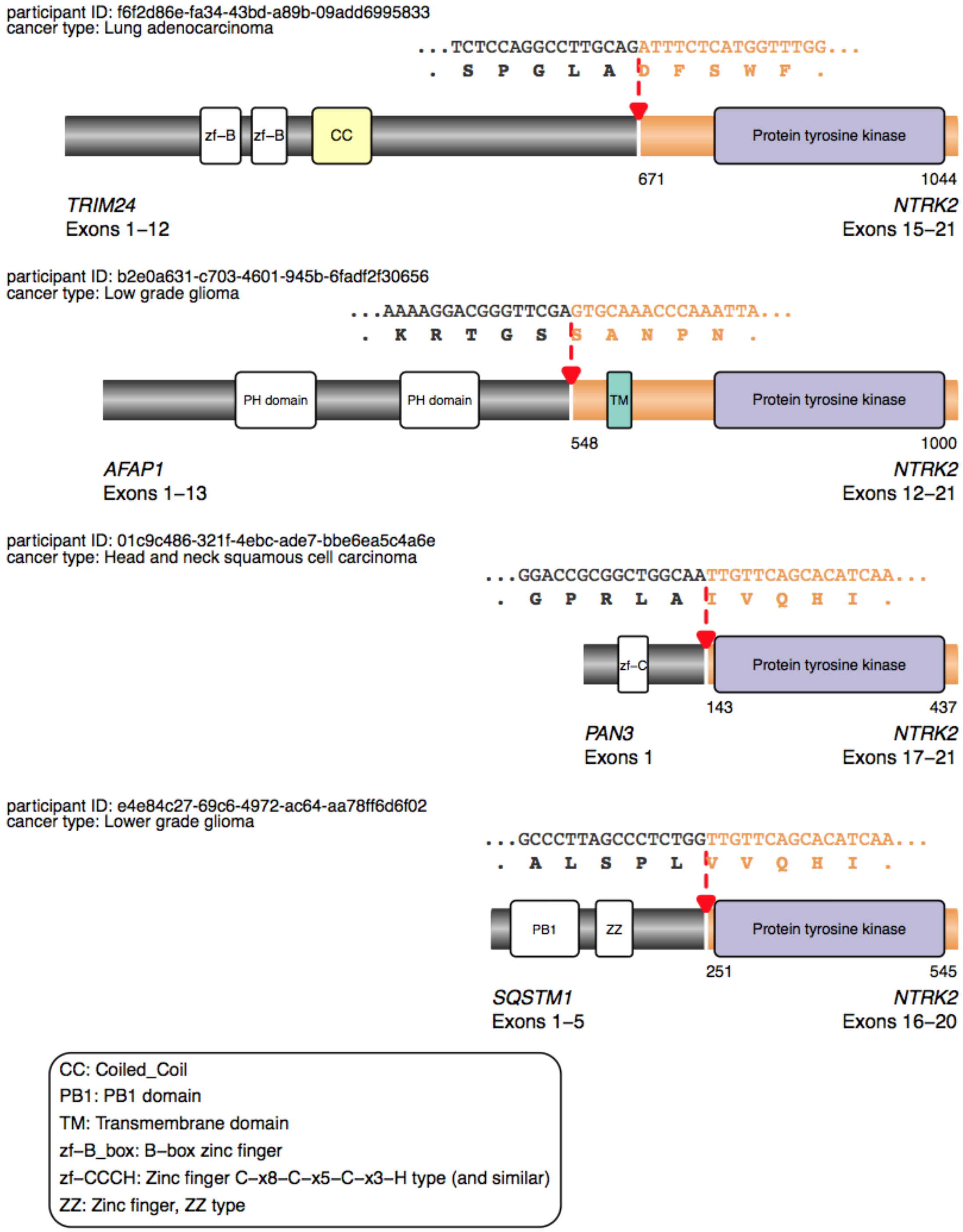

Supplementary Figure 8: *NTRK2* fusions

Protein domains, sample details, cancer type, gene partners, as well as genomic and amino acid sequence are indicated for each predicted kinase fusion proteins. In addition, the protein coordinates of the fusion breakpoints and the total amino acid length of the fusion protein are noted under each protein structure. The protein tyrosine kinase domains are colored in purple, the transmembrane domains (TM) are indicated in teal, coiled-coil (CC) dimerization domains are indicated in yellow, whereas other domains are left in white. Fusion breakpoints are delineated by red arrows.

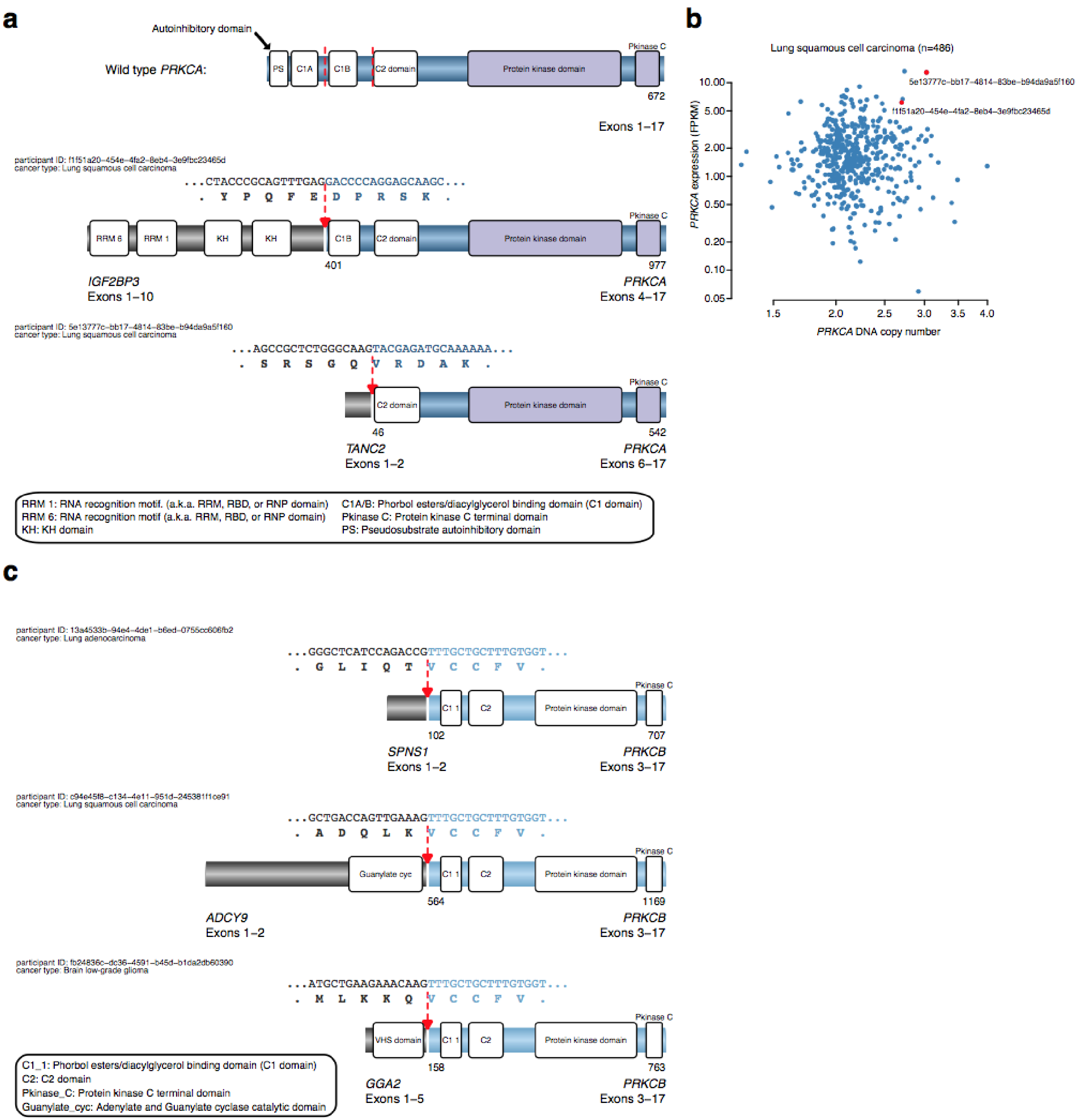

Supplementary Figure 9: *PRKCA*/*PRKCB* fusions

a, Structure of PRKCA wild-type (top) and of the two PRKCA fusion proteins. **b**, Scatter plot of the *PRKCA* copy number versus mRNA expression. Each dot represents a sample with the red dots representing the samples harboring *PRKCA* fusions. **c**, Structure of the three PRKCB fusion proteins.

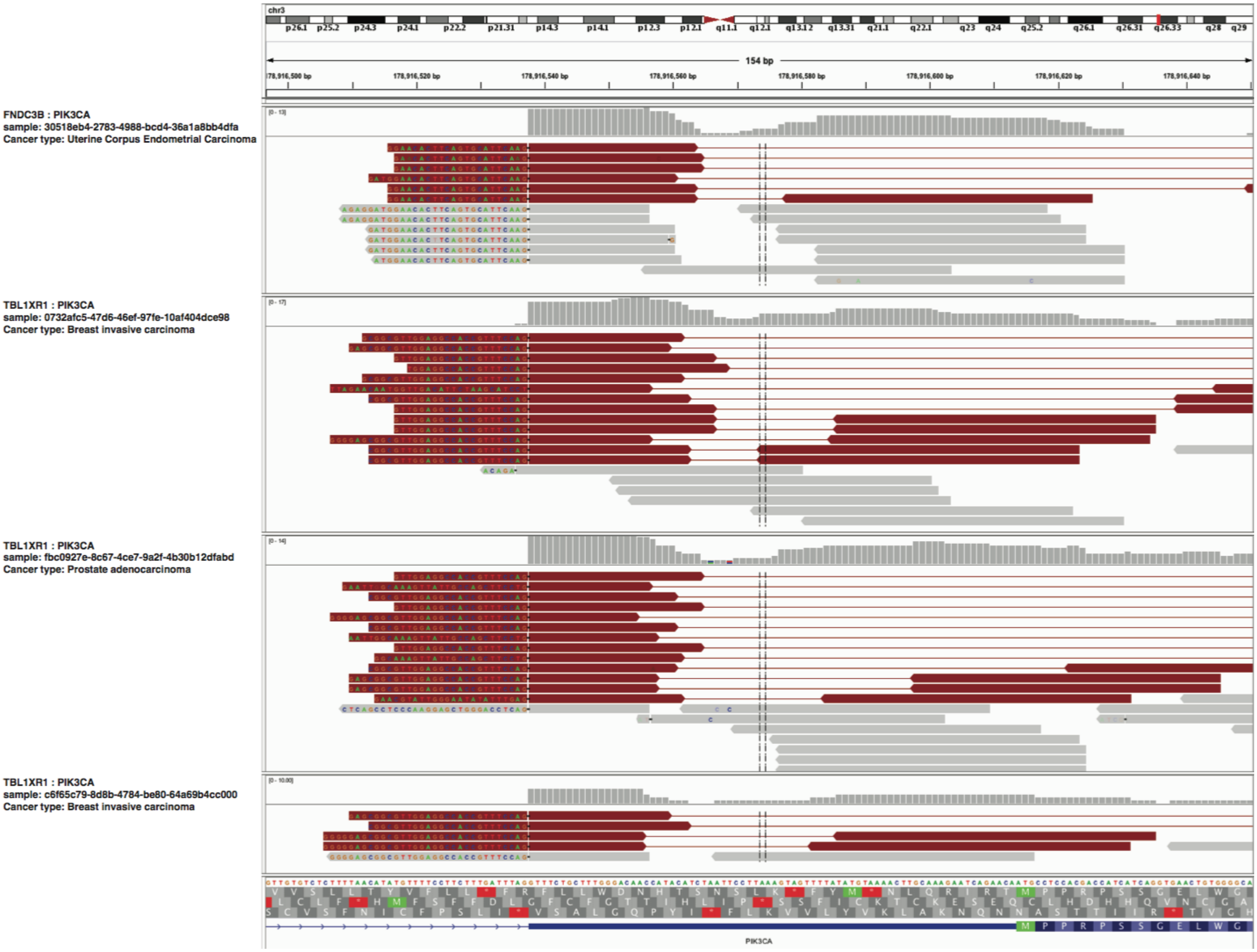

Supplementary Figure 10: *PIK3CA* fusions sequencing reads (1/2)

Sequencing reads mapping to *PIK3CA* (screenshots from the integrative genome viewer) supporting the presence of *PIK3CA* fusions in four TCGA samples. All reads are either chimeric (one end maps to *PIK3CA* and the other end maps to the partner gene) or split between both genes, with soft-clipped bases shown.

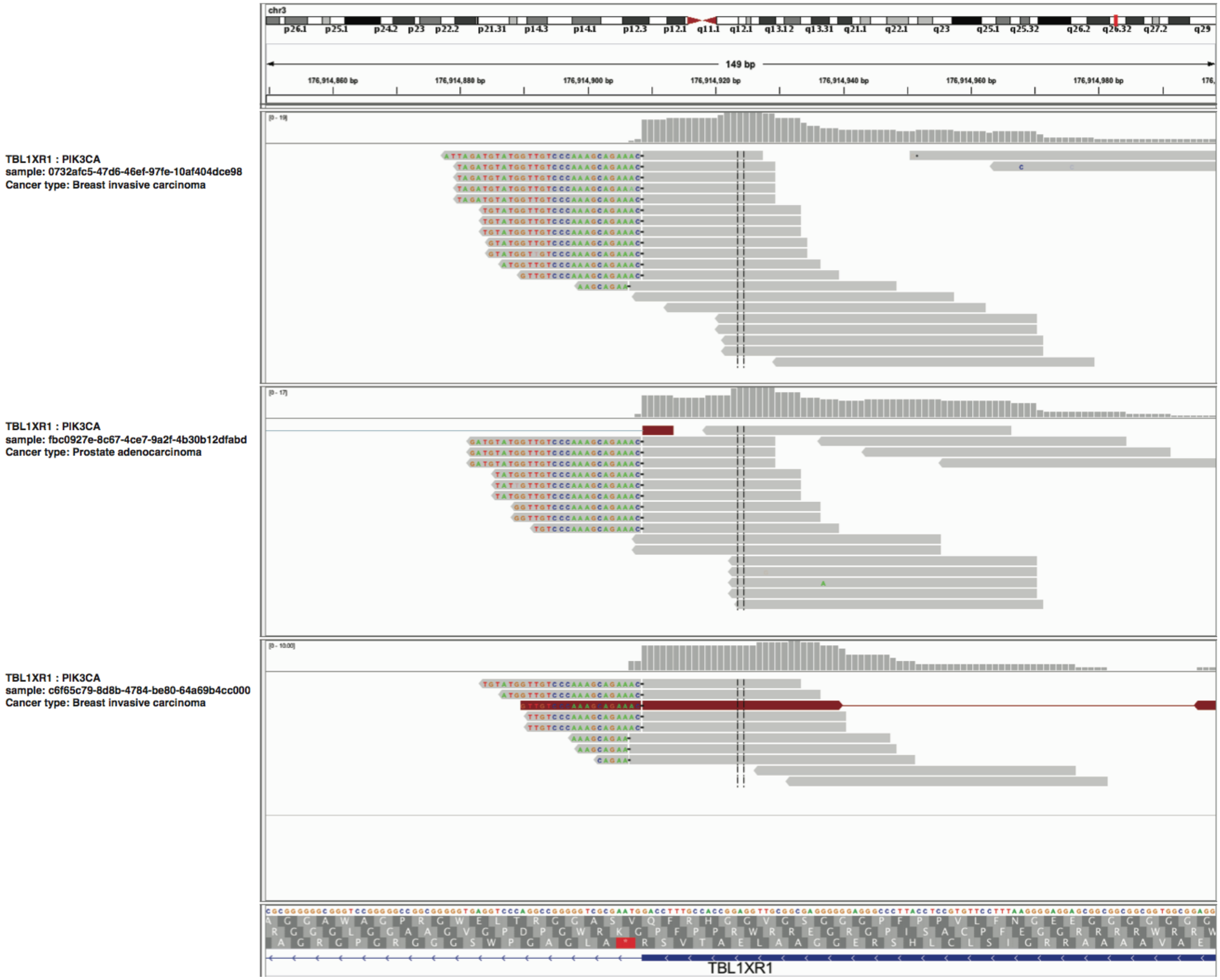

Supplementary Figure 11: *TBL1XR1-PIK3CA* fusions sequencing reads (2/2)
Sequencing reads mapping to *TBL1XR1* (screenshots from the integrative genome viewer) supporting the presence of *TBL1XR1-PIK3CA* fusions in three TCGA samples. All reads are either chimeric (one end maps to *PIK3CA* and the other end maps to the partner gene) or split between both genes, with soft-clipped bases shown.

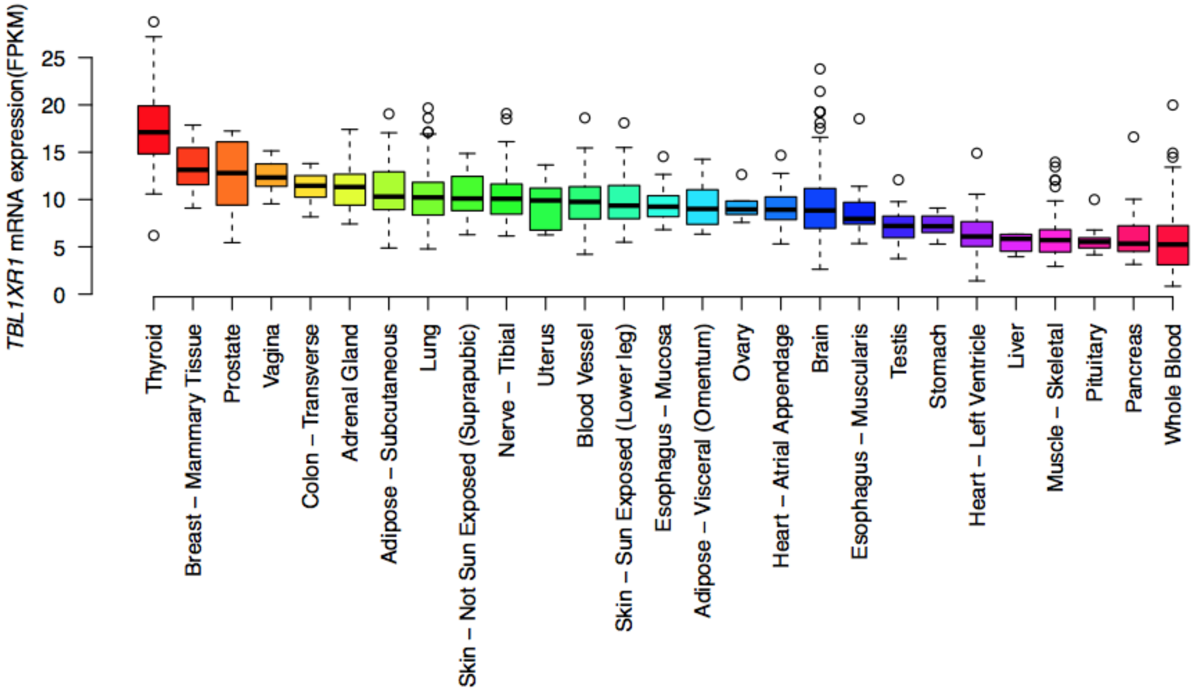

Supplementary Figure 12: *PIK3CA* expression in normal samples.

PIK3CA expression across 1,750 normal samples from the GTEx project, in 27 tissue types, ordered by their median expression (bold horizontal line). Boxplot whiskers extend to the most extreme data point which is no more than 1.5 times the interquartile range from the box (first and third quartiles). Outlier values are indicated with circles.

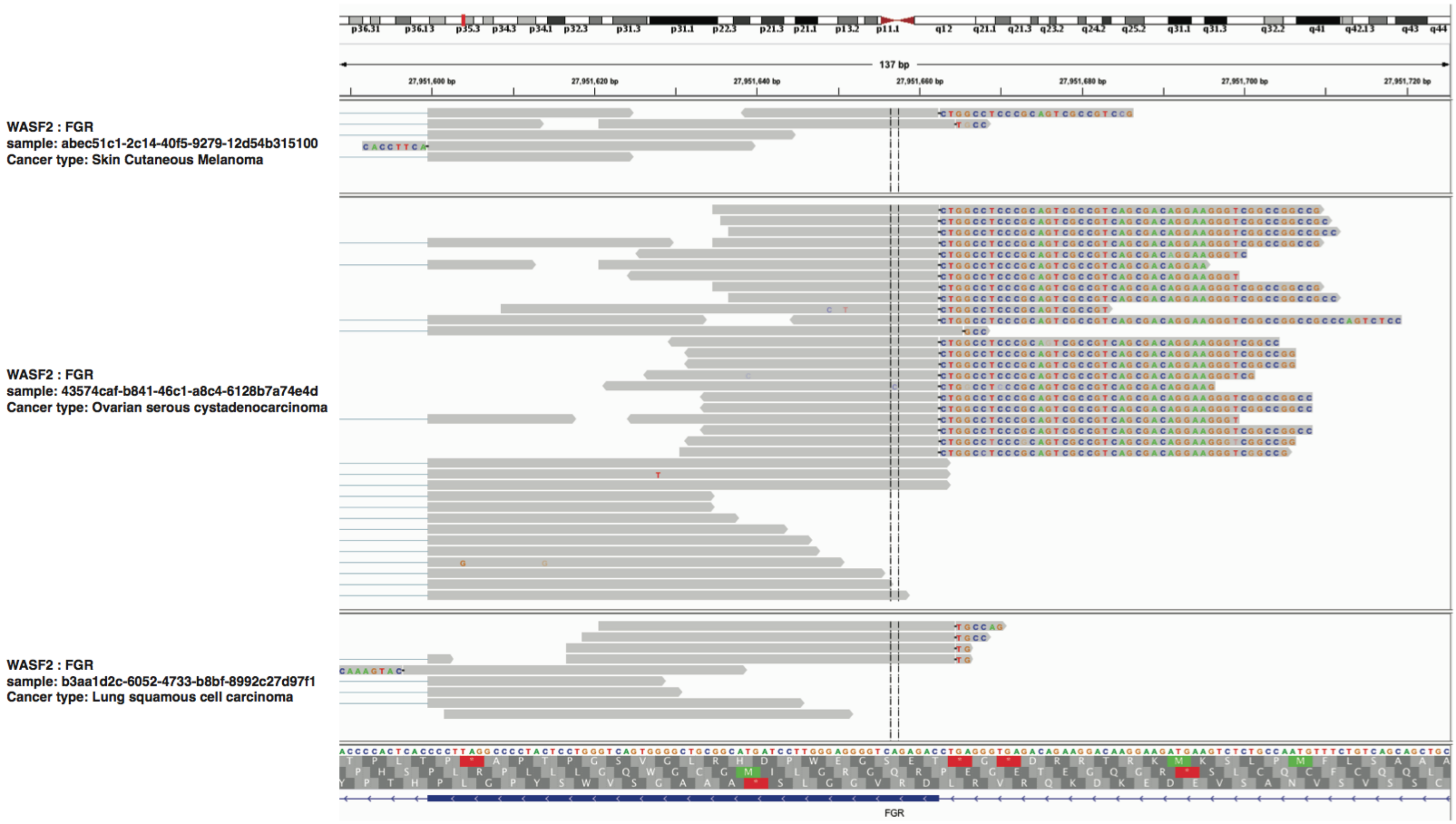

Supplementary Figure 13: *FGR* fusions (1/2)

Sequencing reads mapping to *FGR* (screenshots from the integrative genome viewer) supporting the presence of *WASF2-FGR* fusions in three TCGA samples. All reads are either chimeric (one end maps to *FGR* and the other end maps to *WASF2*) or split between both genes, with soft-clipped bases shown.

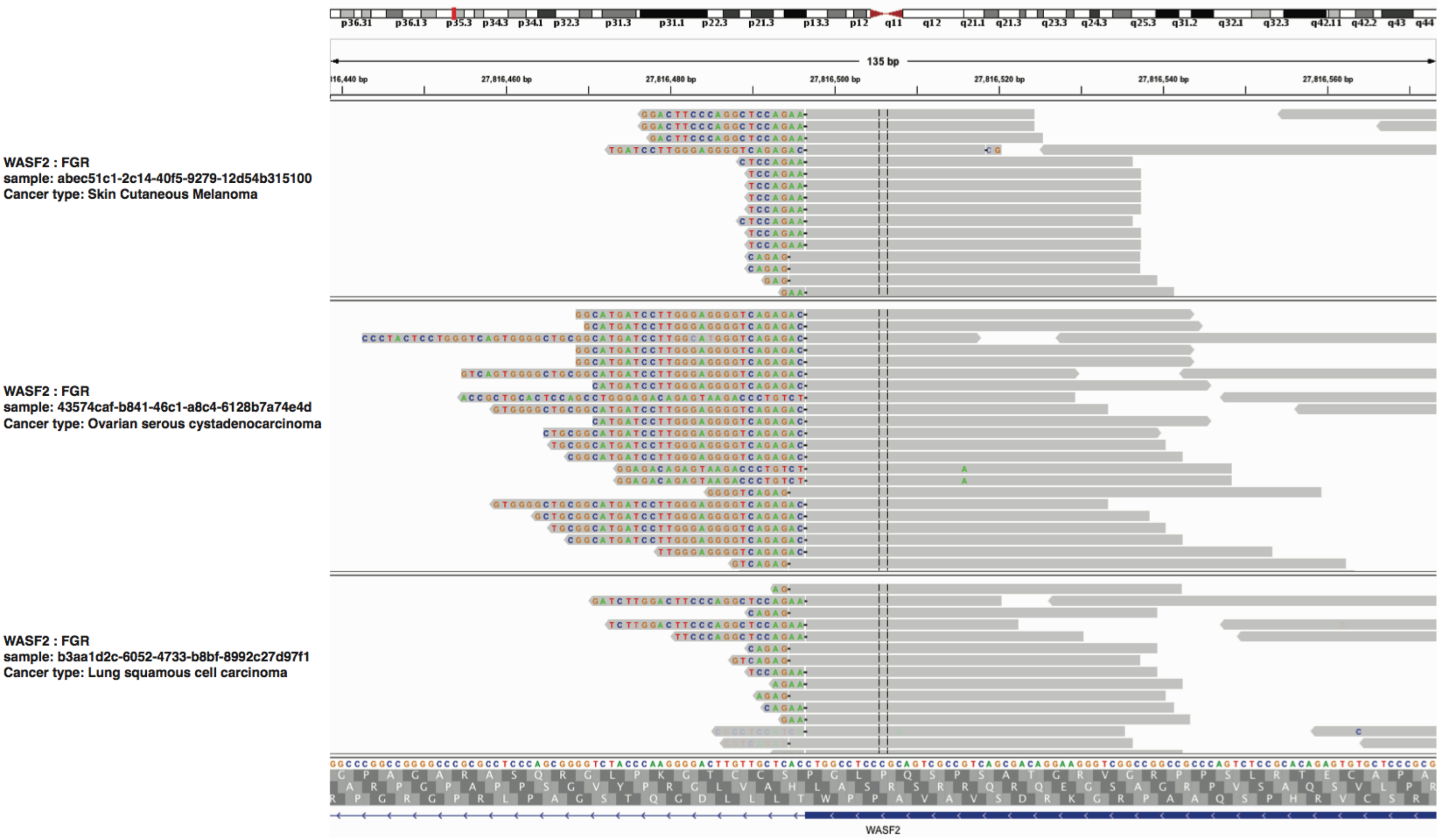

Supplementary Figure 14: *FGR* fusions (2/2)

Sequencing reads mapping to *WASF2* (screenshots from the integrative genome viewer) supporting the presence of *WASF2-FGR* fusions in three TCGA samples. All reads are either chimeric (one end maps to *FGR* and the other end maps to *WASF2*) or split between both genes, with soft-clipped bases shown.

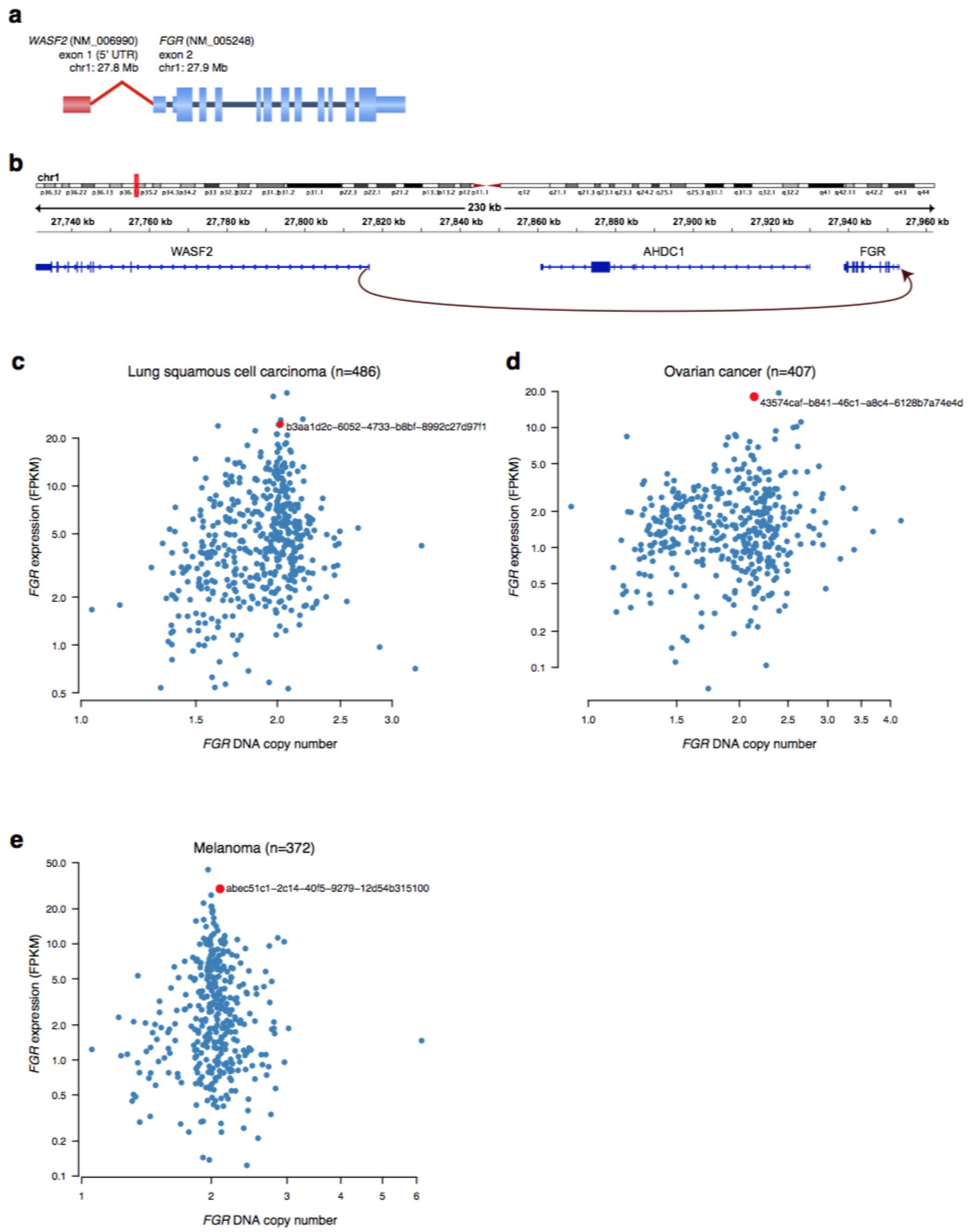

Supplementary Figure 15: *FGR* fusions

a, b, Genomic structure of *WASF2-FGR* fusions. In all cases the entire coding sequence of FGR is present in the predicted fusion sequence. RefSeq IDs, exon numbers, and genomic coordinates are indicated. Exon 1 of *WASF2* (5-prime UTR) is fused to exon 2 of *FGR* (5-prime UTR). **c-e**, Scatter plots of *FGR* DNA copy number versus mRNA expression across all TCGA samples for which both data types are available. Samples harboring a *FGR* fusion are depicted in red (along with TCGA sample ID) and show a high expression of *FGR* relative to the other samples. FPKM: Fragments per kilobase of transcript per million mapped reads.

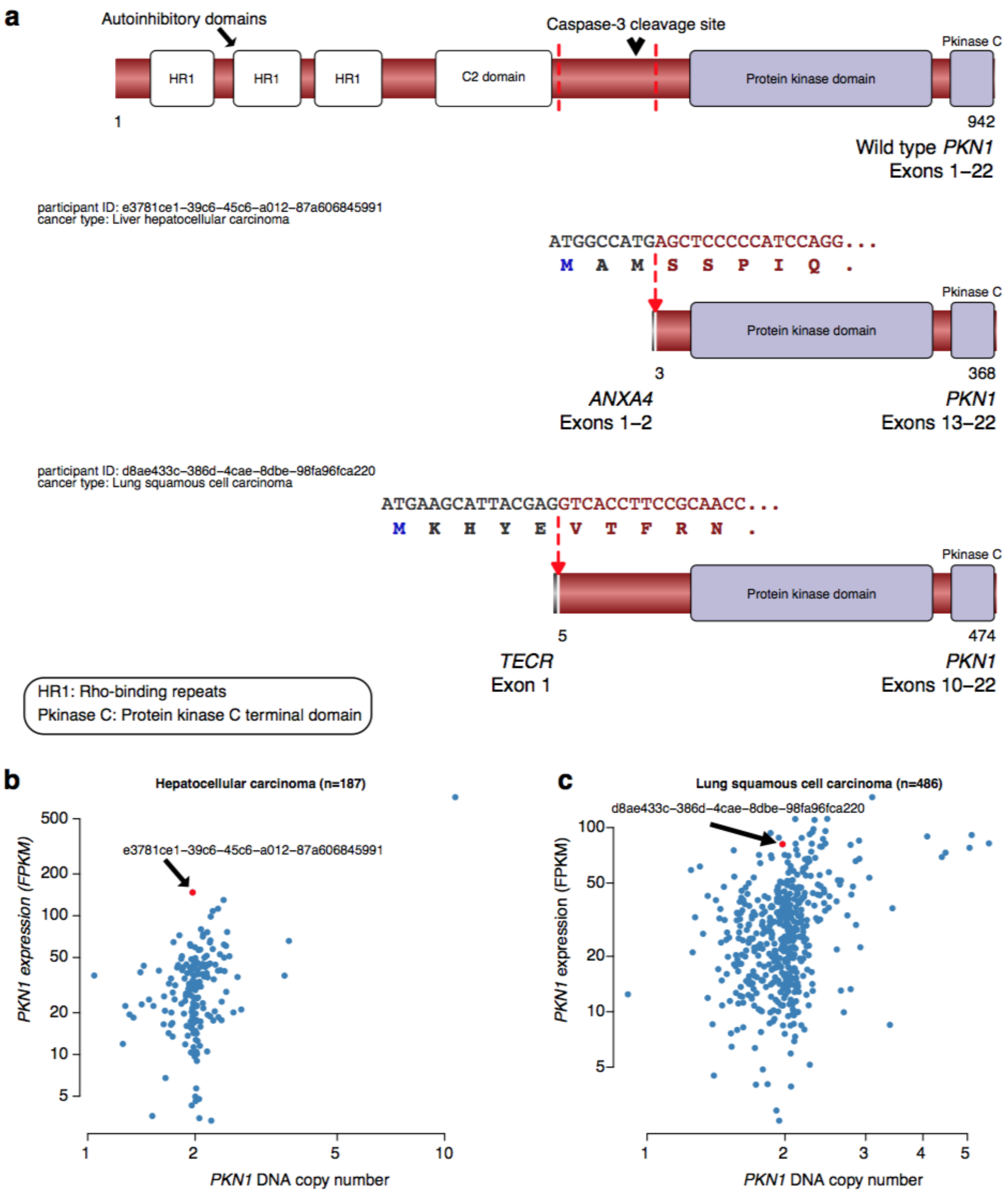

Supplementary Figure 16: *PKN1* fusions

a, Protein structure of PKN1 wild-type (top) and *PKN1* fusions. **b, c**, Scatter plots of *PKN1* DNA copy number versus mRNA expression across all TCGA samples for which both data types are available. Samples harboring a *PKN1* fusion are depicted in red, along with TCGA sample ID.

Supplementary table 1: TCGA cancer types summary

Study Abbreviation	Study Name	n samples
blca	Bladder Urothelial Carcinoma	250
lgg	Brain Lower Grade Glioma	461
brca	Breast invasive carcinoma	1072
coad	Colon adenocarcinoma	286
gbm	Glioblastoma multiforme	157
hnsc	Head and Neck squamous cell carcinoma	411
kich	Kidney Chromophobe	66
kirc	Kidney renal clear cell carcinoma	529
kirp	Kidney renal papillary cell carcinoma	198
lihc	Liver hepatocellular carcinoma	194
luad	Lung adenocarcinoma	513
lusc	Lung squamous cell carcinoma	492
ov	Ovarian serous cystadenocarcinoma	412
prad	Prostate adenocarcinoma	335
read	Rectum adenocarcinoma	91
sarc	Sarcoma	103
skcm	Skin Cutaneous Melanoma	374
stad	Stomach adenocarcinoma	285
thca	Thyroid carcinoma	498
ucec	Uterine Corpus Endometrial Carcinoma	166
